# Photon-efficient optical tweezers via wavefront shaping

**DOI:** 10.1126/sciadv.adi7792

**Published:** 2024-07-05

**Authors:** Unė G. Būtaitė, Christina Sharp, Michael Horodynski, Graham M. Gibson, Miles J. Padgett, Stefan Rotter, Jonathan M. Taylor, David B. Phillips

**Affiliations:** ^1^ School of Physics and Astronomy, University of Exeter, Exeter EX4 4QL, UK.; ^2^ Institute for Theoretical Physics, Vienna University of Technology (TU Wien), A-1040 Vienna, Austria, EU.; ^3^ School of Physics and Astronomy, University of Glasgow, Glasgow G12 8QQ, UK.

## Abstract

Optical tweezers enable noncontact trapping of microscale objects using light. It is not known how tightly it is possible to three-dimensionally (3D) trap microparticles with a given photon budget. Reaching this elusive limit would enable maximally stiff particle trapping for precision measurements on the nanoscale and photon-efficient tweezing of light-sensitive objects. Here, we customize the shape of light fields to suit specific particles, with the aim of optimizing trapping stiffness in 3D. We show, theoretically, that the confinement volume of microspheres held in sculpted optical traps can be reduced by one to two orders of magnitude. Experimentally, we use a wavefront shaping–inspired strategy to passively suppress the Brownian fluctuations of microspheres in every direction concurrently, demonstrating order-of-magnitude reductions in their confinement volumes. Our work paves the way toward the fundamental limits of optical control over the mesoscopic realm.

## Teaser

The strength of 3D optical tweezers is substantially enhanced by shaping the trapping field to fit the particle like a glove.

## INTRODUCTION

The ability to remotely control the motion of small particles with laser light has become a key tool in a diverse range of scientific disciplines, from tests of fundamental physics to applications in the life sciences (*1*–*4*). The most widely used approach is the “optical tweezer,” introduced by Arthur Ashkin in 1986 (*5*), enabling three-dimensional (3D) trapping of microscopic dielectric particles by tightly focusing a single Gaussian laser beam onto the target object. Since their conception, optical tweezers have found a multitude of applications. They have been used to reveal the biomechanics of molecular motors and protein-DNA interactions (*6*, *7*), to drive artificial micromachines (*8*, *9*), to test the fundamental relationship between entropy and information (*10*, *11*), and to suspend nanoparticles as their motion is cooled to the quantum ground state (*12*, *13*).

Given the widespread use of optical trapping, it may seem unexpected that, after more than 30 years, the most commonly used spatial shape of laser beams used to create an optical tweezer is still the conventional Gaussian beam profile suggested by Ashkin (*5*). Gaussian beams do come with many advantages: They are straightforward to create and highly versatile, operating in a broadly similar manner over a wide range of microparticle sizes and shapes (*2*, *14*).

However, this versatility comes at a cost: A Gaussian beam is typically not the optimal shape of light field to tightly trap a given microparticle. This drawback is exacerbated for larger particles whose size is greater than the trapping wavelength. Such objects, which include many types of biological cells, are typically only weakly optically tweezed, suffering from a low trapping stiffness in a Gaussian beam. A straightforward way to overcome this issue is to crank up the laser power: Doubling the intensity of the trapping light, in turn, doubles the trapping stiffness in all dimensions, thus confining a particle more tightly. Unfortunately, increasing the power focused onto the trapped object can lead to a variety of undesirable effects: Excess photons can damage photosensitive biological systems (*15*), heat the particle and its local environment (*16*, *17*), and increase decoherence effects in quantum ground state experiments (*13*).

Here, we explore an alternate paradigm: We demonstrate to what extent it is possible to enhance 3D trap stiffness without increasing laser power, by instead tailoring the spatial profile of the laser beam. The numerous spatial degrees of freedom available render this a highly challenging task from both a computational and experimental perspective. Nonetheless, optical fields have been previously shaped in a plethora of different ways to exert targeted optical forces and torques on microparticles (*18*–*30*). As far as enhancing trapping stiffness is concerned, various beam shapes have been tried in the past (*31*–*36*). An important step forward was taken by Taylor *et al.* (*27*) in 2015, who demonstrated that carefully sculpting the incident optical field can deliver impressive 1D lateral stiffness enhancements of trapped microspheres, by up to a factor of ∼30 compared to conventional optical tweezers of the same power. It is possible to identify globally optimum structured fields that accomplish these 1D stiffness enhancements using eigenvalue-based approaches (*37*–*41*). However, as we show in section S1, these 1D trapping enhancements place no constraints on stiffness in other dimensions and so do not guarantee that a particle is tightly trapped in 3D or even stably trapped at all (*42*).

It is not straightforward to extend such eigenvalue-based strategies to enhance multidimensional optical trapping because the stiffnesses along different directions are not independent. Consequently, the optimum 3D trapping field will not simply be a superposition of optimal trapping fields for the individual axes. Thus, despite multiple decades of research into optical trapping, an understanding of how to calculate the shape of “optimal” 3D optical traps or the level of multidimensional stiffness enhancement that they may deliver has remained out of reach (*42*).

Here, we tackle these difficulties by designing bespoke trapping beams using an integrated multiparameter optimization strategy, guided efficiently to a solution by the information held within the generalized Wigner-Smith (GWS) operator (*40*, *41*). Our approach allows all three dimensions to be considered simultaneously, in terms of both stiffness enhancement and trap stability. We predict that custom-tailored trap shapes can confine microsphere motion to a volume up to 200 times smaller than a Gaussian trap of equivalent power. We find that experimental implementation of such highly optimized trapping fields is extremely sensitive to precise experimental conditions, requiring exact knowledge of both the particle and optical system. Therefore, to render experimental validation of our concept possible, we develop a real-time optimization routine that iteratively adapts the trapping field to the shape of the particle in situ. We experimentally demonstrate order-of-magnitude improvements in how tightly it is possible to squeeze the volume explored by microspheres. Our work establishes that marked gains in 3D optical trapping efficiency are possible by judiciously structuring light fields and presents theoretical and experimental routes to achieve them.

## RESULTS

### Designing bespoke optical traps

When submerged in a liquid, a trapped particle is constantly jostled around by collisions with surrounding molecules that are undergoing Brownian motion. However, regardless of the direction in which the particle is displaced, the laser light of the trapping field is deflected to generate a near-Hookean optical restoring force, pulling the particle back toward its equilibrium position. For small displacements, this force vector is given by **f**^opt^ = −**κΔr**, where **κ** is a 3 × 3–element stiffness matrix encapsulating the translational trapping stiffness in any direction, and vector **Δr** describes the particle’s 3D displacement from equilibrium (*43*, *44*).

**Fig. 1.**
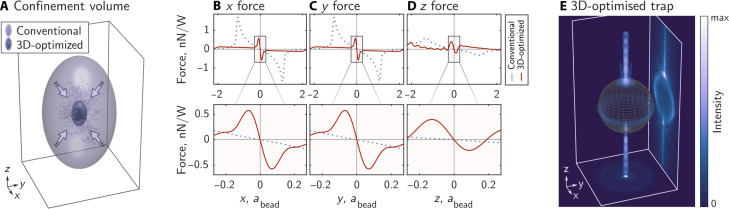
3D-optimized optical trapping. (**A**) Confinement volume (*V*_c_) of an optically trapped microparticle in a conventional (violet) and a 3D-optimized (blue) optical trap, with simulated trajectories of the CoM shown. The confinement volume corresponding to the optimized trap is ∼99 times smaller. (**B** to **D**) Simulated optical force exerted on a microsphere as it is displaced in the *x*, *y*, or *z* directions; shown over a long range (top), and zooming in on the detail at the origin, i.e., trap equilibrium (bottom); see also section S10 for the full force landscape. Here *a*_bead_ is the radius of the microsphere, and the displacement is plotted as a fraction of the microsphere radius. (**E**) Schematic of the intensity of a 3D-optimized optical trap with *xy* and *xz* average intensity projections (each normalized individually, the *yz* projection would be identical to *xz*); see also section S11 for the far-field spatial profile of this trap. In this figure, we model a silica (*n* = 1.45) microsphere of 4.01-μm radius, immersed in water (*n* = 1.326 at the trapping wavelength), and illuminated from the negative *z* direction with circularly polarized laser light of wavelength 1.064 μm, through a numerical aperture (NA) of 1.25.

Thermal motion of the fluid thus drives the particle’s center of mass (CoM) to stochastically explore a small region around its equilibrium position. To quantify the size of this region, we utilize the concept of the confinement volume *V*_c_ (see Fig. 1A), also referred to as the thermal ellipsoid (*45*). The shape of the confinement volume is typically a prolate ellipsoid with its long axis parallel to the optical axis of the trapping beam, reflecting the lower axial trapping stiffness arising from weaker intensity gradients in this direction. *V*_c_ is given by

$$V_{c}=36\pi \sqrt{\frac{k_{B}^{3}T^{3}}{\kappa_{x}\kappa_{y}\kappa_{z}}}$$ (1)

and is such that the probability of finding the CoM inside it is *p* ∼ 0.99. Here, *k*_B_ is Boltzmann’s constant, *T* is the absolute temperature of the surrounding fluid, and κ_*x*,*y*,*z*_ are the eigenvalues of **κ**, which represent the stiffnesses of the optical trap along the principal axes of the thermal ellipsoid (see section S2). Our aim in this work is to find the shape of light fields that reduce the particle’s confinement volume by maximizing all eigenvalues of the stiffness matrix simultaneously.

We begin by investigating the level of 3D optical trapping enhancement possible from a theoretical perspective. We use the T-matrix formalism (see section S3) (*46*–*48*) to model the interaction of a shaped incident light field **u** with a microscopic particle in an optical tweezers setup. The incident field coefficients held in the column vector **u** are defined at the pupil plane of the objective lens, expressed in the Bessel beam basis. Each Bessel beam within this optimization basis is defined by a unique set of indices quantifying the cone-angle, integer value of orbital angular momentum (OAM), and polarization (see section S4), enabling us to explore the consequences of spatially shaping the intensity, wavefront, and polarization of the trapping beam.

We seek to shrink the confinement volume of a trapped particle as much as possible. However, minimizing *V*_c_ directly is not a good strategy because it does not place any requirements on the shape of the confinement volume. This could lead to solutions where the particle is strongly confined in one direction but only loosely trapped in the orthogonal directions. Such behavior is not typically desirable in optical tweezers. We therefore take the approach of constrained optimization, which allows us to specify the aspect ratio of the confinement volume (allowing us to mold it, for example, into a prolate or oblate ellipsoid or a sphere). To achieve this, we set κ*_x_* as the objective function to be maximized, subject to the conditions that κ*_y_* = κ*_x_* and κ*_z_* = κ*_x_*NA/4, an aspect ratio chosen to mimic the shape of the diffraction-limited spot. This method allows us to simultaneously enhance stiffness in multiple dimensions, and we also apply additional constraints to ensure that the particle remains stably trapped; see Materials and Methods and section S5 for more details. A large number of optimization variables *N* (i.e., elements of **u**) is required to shape the light effectively. This makes the problem computationally demanding, with previous examples of numerical 1D trapping field optimization taking on the order of days to converge (*27*, *49*). Therefore, we hone the efficiency of our optimization routine to substantially speed up this process.

The cornerstone of our approach is the GWS operators for optical force (**Q**) and trapping stiffness (**K**) (*40*, *41*, *50*, *51*). These operators reduce the evaluation of force and stiffness along a particular direction to a single matrix equation. For example, along the *x* dimension, we have

$$f_{x}^{\text{opt}}=u^{†}Q_{x}u=u^{†}\left(-iS^{†}\partial_{x}S\right)u$$ (2)

$$\kappa_{x}=u^{†}K_{x}u=u^{†}\left(-\partial_{x}Q_{x}\right)u$$ (3)

where **S** is the scattering matrix describing how the light interacts with the particle (i.e., which incident light modes are scattered into which outgoing modes), ∂*_x_* indicates a partial derivative with respect to the *x* position of the particle, and ^†^ indicates a conjugate transpose. Equivalent expressions can be written down for any other degree of freedom, including rotations. Crucially, **Q** and **K** only encapsulate properties of the particle and are independent of the incident field. As such, they only need to be evaluated once, before the optimization routine commences, substantially cutting down on computational time. Expressions for *f*^opt^ and κ are also readily differentiable with respect to **u**, providing access to analytical expressions for gradients and Hessians, thus further speeding up the optimization. See sections S1 and S6 to S8 for more details on **S**, **Q**, and **K**, including our derivations of analytical expressions for calculating the derivatives in Eqs. 2 and 3. This approach reduces the timescale for a single trap design from days to minutes (see section S9), allowing us to explore the enhancements achievable over a wide range of different particles.

### Theoretical trapping enhancements

An example of one such optimized trap can be seen in Fig. 1, here designed for a microsphere of diameter ∼8 times larger than the laser wavelength. Our modeling shows that the achieved confinement volume is ∼99 times smaller than that of a conventional optical trap carrying the same power, as seen in Fig. 1A. Figure 1 (B to D) presents optical force curves of the optimized trap, indicating that the trapping stiffness has been increased by factors of ∼22, ∼22, and ∼19 in the *x*, *y*, and *z* directions, respectively. In Fig. 1E, we see that the optimized optical field forms a high intensity ring around the equator of the microsphere, as well as tracking around the inside of its surface (most clearly observed in the *xz* projection), in stark contrast to a conventional Gaussian beam that typically focuses at the particle’s center. This optimized field shape can be understood by considering that momentum transfer between laser light and a trapped particle can only take place where there is a spatial gradient in refractive index, i.e., at the interface between the particle and the surrounding medium (*52*) (for a non-absorbing, homogeneous, and isotropic particle, as in this case). Thus, our optimizer achieves 3D trapping stiffness enhancement by boosting the intensity of light at the particle’s boundaries, as well as simultaneously ensuring a stable trap by balancing momentum transfer due to specular reflections from the particle. For more examples of 3D renderings of optimized traps, see movie S1.

**Fig. 2.**
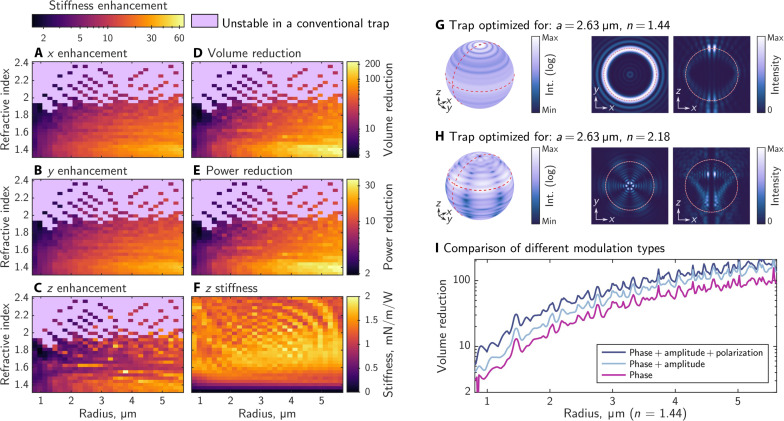
Exploring 3D optical trap enhancement as a function of microsphere size and refractive index. (**A** to **C**) Simulated trapping stiffness enhancement (when compared to a conventional optical trap) achieved simultaneously in each dimension. (**D**) Volume and (**E**) power reduction resulting from these stiffness enhancements. Lavender shaded areas indicate particles which cannot be stably trapped along the axial direction in conventional optical tweezers, and so an enhancement ratio cannot be computed for these particle parameters. Note that (A) to (E) are plotted on a logarithmic scale to better reveal detail. (**F**) Simulated *z* stiffness achieved with our optimized traps; note that, by design, the *x* and *y* stiffness is 3.2 times greater than the *z* stiffness (see section S5) and follows a virtually identical trend. (**G** and **H**) Examples of 3D-optimized trap intensity distributions; plotted on the surface of the particle and at cross sections indicated with the dashed red lines on the surface plots (the white-red dashed outlines indicate the edges of the particle). (**I**) Comparison of volume reductions achieved with different far-field modulation strategies. The same simulation parameters as in Fig. 1 were used.

Figure 2 shows the theoretical optical trapping enhancements possible for 900 scenarios involving spherical particles of different sizes (in the range of 0.8 to 5.7 μm in radius) and refractive indices (*n* = 1.33 to *n* = 2.4). We optimize the phase, amplitude, and polarization of *N* = 814 spatial modes within each trapping beam. We compare the theoretical performance of these optimized optical traps against a conventional optical trap of the same numerical aperture (NA) in two ways: We show the directional stiffness enhancement as the factor of improvement in the eigenvalues of **κ** in Fig. 2 (A to C) and the confinement volume reduction $V_{c}^{\text{rel}}$ as the factor of improvement in *V*_c_ in Fig. 2D.

Our simulations indicate that it is, in theory, possible to achieve very substantial 3D trapping enhancements, in some cases of up to a 200-fold reduction in the confinement volume, and with stiffness enhancements exceeding ∼20 in all dimensions simultaneously for a broad range of microsphere sizes and refractive indices. Equivalently, these reductions in particle confinement volume can be translated into improvements in relative trapping efficiency η^rel^: the factor of the reduction in trapping power needed to hold a particle as tightly as a conventional optical trap, where $\eta^{\text{rel}}={\left(V_{c}^{\text{rel}}\right)}^{2/3}$ (see section S12 for derivation). In Fig. 2E, we see that η^rel^ is over a factor of 10 for a wide range of particles, predicting that the same trapping performance as a conventional optical tweezer can be achieved using less than 1/10th of the laser power. In addition to this, the peak intensities on particles are typically reduced by more than a factor of η^rel^ because the optimized traps spread out the light across the particle, unlike a conventional trap where all incoming power is focused into a single spot. This offers a potential additional advantage when working with photosensitive specimens. We find that these enhancements are highly repeatable, irrespective of the choice of initial values used for the complex optimization variables **u** (see section S13). This suggests that we may be finding solutions that are close to the global optimum, given the set of constraints that we have enforced.

A notable feature of our method is that it predicts an extension of the range of particle parameters over which optical trapping is possible: Conventional optical tweezers fail to create stable *z*-equilibria for high–refractive index particles [unless antireflection coatings are engineered (*53*)], while we see in Fig. 2F that stiff and stable optical trapping becomes possible in this high-index regime by shaping the structure of the trapping field (see also section S14 for conventional trap stiffness). Another dominant trend in these results is that large particles with low refractive indices benefit the most. The enhancements are lower for small dielectric particles (<1-μm radii, i.e., smaller than the trapping wavelength of 1.064 μm) because the diffraction limit restricts the extent to which the trapping light can be shaped within the footprint of the particle.

Figure 2 (G and H) shows two representative examples of the intensities of these bespoke optical traps on the surface and in transverse and axial cross sections through the particles (also see section S15 for additional intensity cross sections). For the majority of the particles that we simulated (92%), the optimized trapping fields follow the circular symmetry of the microspheres themselves and form intensity distributions that are rotationally invariant about the optical axis (Fig. 2G, surface intensity plot), typically forming rings of intensity in any transverse plane (Fig. 2G, *xy* cross section). In the remaining 8% of the cases, we observe that the optimized trapping light concentrates into several high intensity “hot spots” on the surface of the particle (Fig. 2H), a feature more typical for particles of high refractive index. The optimized trapping fields are typically linearly polarized in the transverse plane through the middle of the particle, with a polarization direction that may rotate in the plane when circumnavigating the particle (see section S16 for examples).

Driven by typical experimental capabilities, we also investigate enhanced optical trapping when far-field spatial polarization shaping is not available, as well as when using phase-only beam shaping (see section S17 for how we reformulate our numerical optimization routine to incorporate these constraints). Figure 2I shows confinement volume reductions achieved with the three modulation types for a range of particle sizes of the same refractive index. Here, fixing the polarization decreases the volume reduction by 1.1 to 2.2 times, and eliminating amplitude control drops the volume reductions further by 1.1 to 1.7 times (with one outlier where the volume reduction is 5.2 times smaller for a particle with 0.85-μm radius). We note that the peaks and valleys in the confinement volume reduction data are inherited from the Mie-resonances characteristic to conventional optical tweezers. We present the comparison across the full dataset of 900 particles in section S18. In the case where the far field is uniformly polarized, we used circularly polarized light to preserve the circular symmetry of the focus in a high NA system, although we observed that, for high–refractive index particles, linearly polarized light performs better; see section S19 for details. Overall, the optimized traps using phase-only modulation still offer substantial trapping enhancements, yielding confinement volumes of up to 140 times smaller than offered by a conventional optical trap and thus showing promise for experimental implementation in the most widely used (i.e., phase-only) wavefront shaping configuration.

### Live-optimized 3D optical trapping

**Fig. 3.**
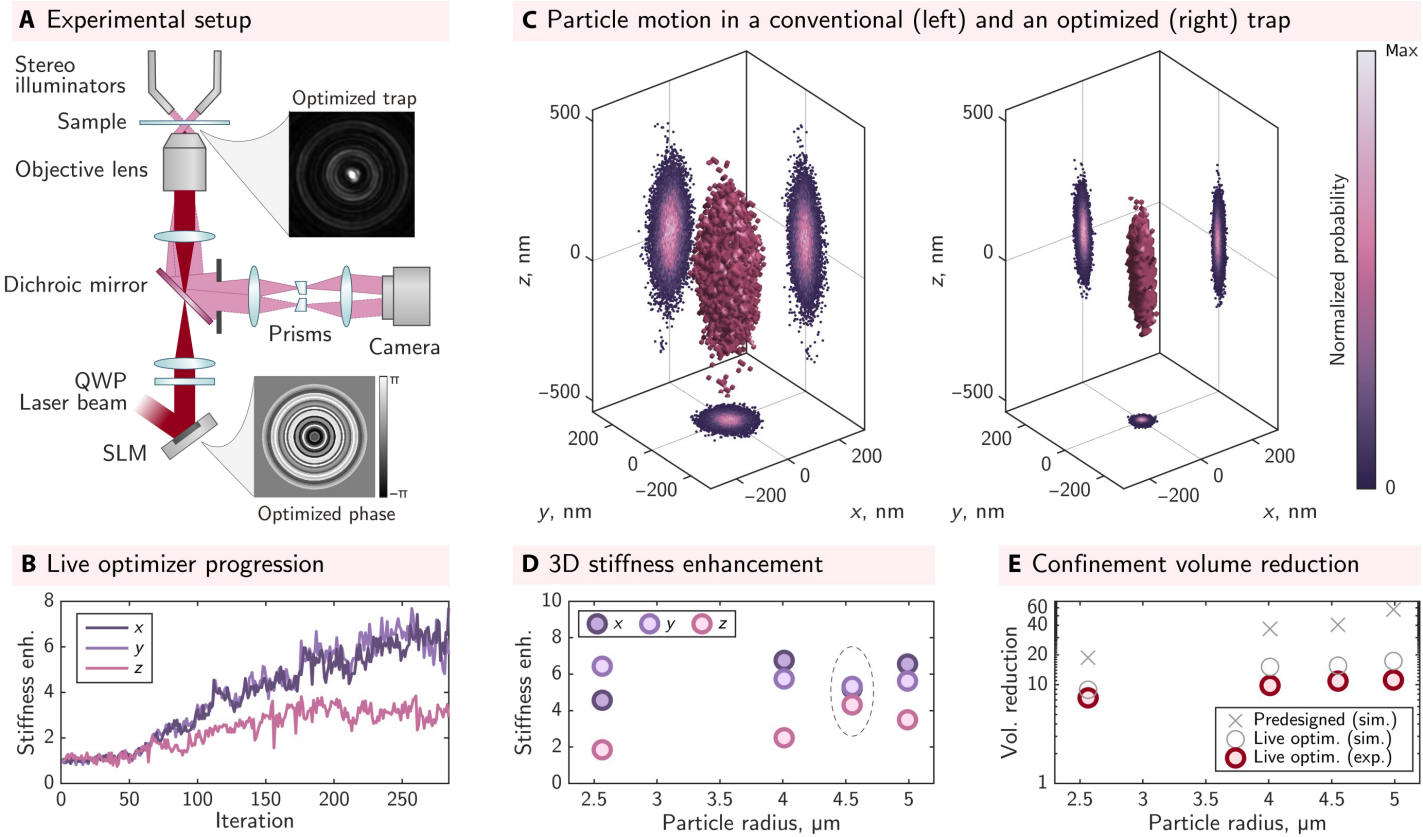
Experimental results of live-optimized optical trapping. (**A**) Schematic of the holographic optical tweezers system used for the experiments. The top inset shows an example image of an experimentally optimized trap. QWP, quarter-wave plate. (**B**) Experimental data showing the thermal volume explored by a 5-μm-radius silica microsphere, with projections of 2D position probability distributions of the CoM. (**C**) Example of the progression of the live optimizer in experiment. (**D**) Typical stiffness enhancements obtained for a range of particle radii (as quoted by manufacturers) and (**E**) corresponding volume reduction factors. See section S25 for a discussion of errors.

Encouraged by the predictions of our fully vectorial 3D model, we now investigate the experimental implementation of optimized 3D optical trapping. We use holographic optical tweezers and a 3D particle tracking platform, shown schematically in Fig. 3A. The trapping field is shaped with a liquid crystal spatial light modulator (SLM), conjugated with the pupil of the objective lens. The 3D motion of trapped particles, immersed in water, is tracked in real time (at up to 1 kHz) using high-speed stereo-microscopy, delivering nano-metric precision in three dimensions (*33*, *54*). See Materials and Methods for more details.

A number of specific challenges arise when seeking to apply a numerically predesigned trapping field in a real-world experiment. Commercially available colloids exhibit a statistical variation in size, and our modeling shows (section S20) that the optimal trap shape is highly dependent on the exact physical properties of the target particle. The volumetric trapping fields must also be generated with extremely high fidelity because the mechanical response of the particle is highly sensitive to small variations in the applied trapping field. The creation of predesigned fields is influenced by factors including the imperfect response of the SLM (*55*, *56*), the precise transfer function of the objective lens (*57*–*60*), and the configuration of the oil-glass-water interfaces that light passes through on its way to the target particle (*61*), even after in situ aberration correction is performed with the SLM (*62*). The combined effect of the above factors means that using predesigned optimized traps is very challenging.

Section S21 gives details of our attempts to implement our predesigned optical traps experimentally. We conducted a variety of tests: For each particular pre-optimized trap design tested, we cycled through approximately 100 designs searching over a local range of microsphere sizes (centered on the average quoted size) and a local range of scaling factors of the pattern placed on the SLM (centered on the theoretically calculated scaling factors). However, these initial endeavors did not yield substantial enhancements, the best result reducing the confinement volume by a factor of ∼4 instead of the anticipated ∼36. We attribute this to a lack of knowledge of the precise sizes of the target microspheres themselves and the fact that our design algorithm relies on an approximate model of the vectorial optical transfer function of our optical tweezers system and sample chamber. This transfer function is very challenging to accurately characterize in a high NA optical system but, ideally, should be incorporated into the trap design protocol.

We therefore devised an experimental strategy compatible with these challenges. We take inspiration from the field of wavefront shaping that has emerged as a powerful way to optimize coherent light transport in unknown complex scattering environments (*63*–*65*). Instead of predesigning optical traps using a simulation of our experiment, we implement a live iterative optimization routine that automatically tailors the trapping field to the (unknown) properties of a target particle. This optimization is guided by real-time measurements of the 3D trapping strength, inferred from the stochastic trajectory of the particle. Crucially, our approach does not require a priori knowledge of the properties of the target particle or the optical characteristics of optical tweezers platform itself because the optimization is based purely on experimentally measured metrics. Guided by our simulations that show high enhancements from circularly symmetric traps, we define a minimal search space of circularly symmetric spatial modes: We optimize the relative phases of a set of ∼30 concentric annuli displayed on the SLM, which correspond to a set of Bessel beams of differing cone angles illuminating the particle. See Materials and Methods for a detailed description of our algorithm.

Figure 3B shows a typical progression of the live optimizer: The stiffness in all three directions gradually increases, reaching a plateau after a few hundred iterations. Figure 3C displays measured point clouds representing the confinement volume explored by the CoM of a 5-μm radius silica microsphere trapped in water using conventional optical tweezers (left) and an optimized trap (right). We see that the thermal ellipsoid has shrunk in all dimensions, in this case, reducing in volume by a factor of $V_{c}^{\text{rel}}\approx 13$ . In terms of their shape, the experimentally live-optimized traps project intensity outward from the center of the particle into rings, in a similar manner to the predesigned traps. This can be seen in the inset in Fig. 3A as well as in section S22.

We validate our live-optimization strategy on silica microspheres ranging in size from 2.5 to 5 μm in radius. Figure 3D shows examples of trapping stiffness enhancements for these different microsphere sizes. In all cases, we observe that stiffness enhancement factors in the *x* and *y* dimensions (∼5 to 7) exceed those in the *z* dimension (∼2 to 4). Cases where the *z* stiffness enhancement is larger result in a concomitant reduction in the *x* and *y* enhancements, as highlighted for the trap optimization targeting the 4.5-μm radius microsphere (points enclosed by a dashed ellipse).

Figure 3E shows the reduction factors of the 3D confinement volume ( $V_{c}^{\text{rel}}$ ) for each microsphere size. Our experimentally optimized traps deliver about an order-of-magnitude reduction in the confinement volume across all tested particles. This corresponds to an improvement in trapping efficiency by a factor of ∼5, i.e., the same 3D trapping stiffness as conventional optical tweezers can be achieved using only ∼20% of the original laser intensity. Traps optimized for larger microspheres tend to yield higher enhancements, consistent with our expectations from theory and simulations. For the smallest and largest microspheres, we perform five optimization runs on different particles within the sample to demonstrate the repeatability of our live-optimization strategy (see section S23), despite variation in particle size and the presence of substantial measurement noise.

## DISCUSSION

We have experimentally demonstrated the potential of optical traps specifically customized to enhance microparticle confinement in all three dimensions simultaneously. For the range of silica microspheres that we consider, our modeling of ideal predesigned traps predicts that it is possible to use phase-only optimization to suppress confinement volumes by factors of $V_{c}^{\text{rel}}\approx 20\;\text{to}\;60$ (see Fig. 3E). Our proof-of-principle experiments reach ∼20 to 40% of these values ( $V_{c}^{\text{rel}}\approx 6\;\text{to}\;13$ ). We attribute these differences mainly to the reduced search space available to the live optimizer due to the need to maintain trap stability in all steps throughout the optimization pathway, something that the predesigned trap optimizer does not need to take into account. We observe that the final live-optimized traps seem to deliver light to the edges of the particle less effectively, featuring more light close to the optical axis of the beam, and so can be loosely interpreted as a superposition of a focus concentrating light to the center of the particle and a structured beam that diffracts light to the particle’s edges to increase the trapping stiffness. This would explain why they offer smaller stiffness enhancements than the predesigned traps because less of the available intensity is concentrated at the refractive index interface between the microsphere and the surrounding medium where momentum can be exchanged.

To better understand the limitations of our live-optimization strategy, we simulate its performance when stiffness measurements are subjected to the level of noise found in our experiments (this noise arises through the need to estimate the stiffness, at each iteration, from a Brownian trajectory captured over a finite length of time; for experimental noise analysis, see section S24). Figure 3E shows the anticipated reduction in confinement volume based on these simulations (gray circles), which indicate that the expected trapping enhancements achieved using live optimization are consistently lower than those achievable with predesigned optical traps. By comparison, our experiments reach ∼60 to 90% of the simulated volume reductions using this method. The most significant difference between these simulations and our experiments is the lower experimental *z* enhancement. We speculate this is likely due to a combination of factors: (i) the experimental particle tracking precision is lower in the axial direction, meaning that the error in the axial stiffness measurements is higher, as discussed in section S25; (ii) the relaxation time of the bead’s motion in the axial direction is longer, further increasing the relative error in axial stiffness estimation for measurements conducted over a given time window; and (iii) the fact that it is typically the rays traveling at high angles with respect to the optical axis, which result in axial restoring forces, and these rays may suffer more losses and aberrations when propagating through the high NA objective lens; therefore, we have lower-fidelity experimental control over these components of the field. Other differences between our simulations and experiments are the real-world SLM diffraction losses that occur as the complexity of the displayed patterns increases (see section S26), and the presence of minor system aberrations that break the circular symmetry of the trapping field, neither of which feature in our simulations.

Our experimental procedure is reminiscent of aberration correction techniques that iteratively optimize a pupil function to achieve a diffraction-limited focus in the presence of optical aberrations, typically guided by image quality metrics (*66*). Despite this similarity, our approach is doing something very different: Driven by the motion of the trapped particle itself, light is redistributed out of the focus to the edges of the particle, shown by the presence of intensity rings in images of the final optimized beams (Fig. 3A and section S22). To further evidence that our algorithm is achieving more than simply correcting aberrations in our optical system, we reran the experiment when constrained to search over a basis of low-order Zernike modes, which led to a negligible improvement in the trapping stiffness in any dimension. Last, we note that the levels of stiffness enhancement that we observe (several hundreds of percents) are far beyond the levels previously shown due to aberration correction in optical tweezers (several tens of percents) (*67*).

Our proof-of-principle optimization experiments, performed in an overdamped liquid environment, are slow due to the time taken to accurately sample the stiffness of the trap at each iteration (see Materials and Methods). This time could be decreased by increasing the laser power, thus reducing the relaxation time of the particle. If applied to trapping in under-damped environments, such as air or vacuum, then this sampling time would be markedly reduced (*68*), potentially allowing a larger number of modal components to be optimized. Furthermore, in the future, it may be possible to develop faster and more sophisticated live-optimization algorithms with improved resilience to noise (*69*) or using rapid measurement schemes revealing how to improve all modal components of the beam simultaneously (*70*). We also anticipate that it will become feasible to directly experimentally deploy pre-optimized traps following high-fidelity system and sample calibration, so that the trap design algorithm accurately captures the capabilities of the experimental platform. One way of achieving this would be to directly measure and thus optimize the vectorial optical field at the focal plane of the objective lens (*71*). Section S27 shows a direct comparison of our experimental results with all simulated optimization approaches, highlighting the future potential of such customized 3D optical trapping.

We now consider the advantages and trade-offs of enhanced optical trapping more broadly. Our optimized trap design algorithm is efficient and versatile: capable of full-field or phase-only optimization, compatible with microscopes of any NA up to a solid angle of 4π steradians [thus including counter-propagating dual-beam trapping (*72*, *73*)], and can be readily extended to optimize beams with spatially varying polarization and to multispectral light control. Our strategy also allows the aspect ratio of the confinement volume to be freely tuned (as shown in section S5) (*33*). Additional optimization criteria, such as prescribed optical forces or optical torques about any axis, can also be specified using the GWS operators (*29*, *30*, *41*).

Here, we have focused on the discovery of bespoke traps for homogeneous and isotropic microspheres. However, these design techniques can be applied to optimize traps for particles of arbitrary geometry by first pre-calculating the shape’s T-matrix; in these cases, the optimized trapping fields will depend upon particle orientation. We have shown how high–refractive index particles, previously considered “untrappable,” have the potential to be stably held using appropriately shaped beams. Furthermore, our design algorithm can also generate previously unknown forms of optimized “bottle-beam” or “dark” optical tweezers (*19*, *74*), capable of stably holding objects of lower refractive index than the surrounding medium. Current dark trap design methods struggle with extended low-index particles that are substantially larger than the diffraction limit (*75*), a challenge our optimizer can overcome, as shown in section S28.

It is important to note that trapping stiffness enhancements tend to come at the expense of a reduction in the energy barrier preventing a particle from escaping the trap, as also observed previously for 1D optimized traps (*27*). This effect is noticeable in Fig. 1 (B to D), where the stiffness at the origin is many times higher for the optimized trap, yet the trapping range and the maximum restoring force are reduced. Over the range of particles that we studied, the optimized traps generated using full polarization, amplitude, and phase control have their trapping range (i.e., the region of space over which the particle remains trapped) reduced by a factor of up to ∼40 in the *x* and *y* dimensions when compared to conventional optical tweezers. Along the *z* dimension, the trapping range can be reduced by up to ∼19 times (although we find that some high–refractive index particles actually see an up to ∼4 times improvement). Similarly, the maximum restoring force in the optimized traps is reduced by up to ∼6 times in the transverse plane, while, in the axial direction, it is reduced by up to ∼7 times (although we find some cases where it is increased by up to 200 times); see section S29 for the full dataset. In practice, this means that the beam must carry enough power to counteract the thermal motion of the particle and prevent it from “jumping out” of the trap, and this effect becomes more acute for traps optimized for particles of high refractive index (see section S30).

Our concept relies on being able to shape the incident field across the footprint of the target object. Therefore, dielectric particles of a diameter close to the diffraction limit and smaller do not benefit from marked 3D stiffness enhancements over conventional optical tweezers, although we note that sub-diffraction–limited super-oscillating beams may offer opportunities for tightly trapping smaller particles (*36*). In addition, lossy metallic particles cannot presently be treated by our fast optimizer in its current form, due to its reliance on flux conservation. Recently, efficient single-parameter numerical optimization of force or torque on metallic nanoparticles was demonstrated (*76*), pointing to a way forward in this regime.

Last, we note that holographic beam shaping has previously been used to adapt the 3D intensity of light to match the shape of trapped particles (*77*), an approach capable of holding irregularly shaped objects at desired positions and orientations, which can be viewed as optimizing the trapping stability. Our concept is fundamentally different; we aim to optimize trapping stiffness, which entails shaping light fields to create high intensity gradients on the boundaries of a homogeneous particle rather than projecting uniform intensity throughout its volume. Recent work has also begun to explore ways to identify regions of high–refractive index gradients within large inhomogeneous particles to exert higher optical forces by focusing light onto these areas (*78*).

In summary, we have shown that there is plenty of scope to enhance optical trapping through wavefront shaping and that order-of-magnitude improvements in 3D particle confinement are now within reach. This concept represents a passive alternative to active feedback-based position clamping methods, which suppress the motion of an optically trapped particle by rapidly tracking its motion and reactively translating the trap position to continuously maximize the optical restoring force returning the particle to its equilibrium position (*79*, *80*). In the future, we envision that a union of customized light fields applied in concert with specifically engineered microparticles will lead to ultra-stiff and high-force optical traps with specialist capabilities (*20*, *52*, *53*, *81*). The areas of optical trapping that we expect to benefit most from these advances are those that require ultraprecise manipulation of microparticles or feature samples intolerant of high optical intensities. Examples include optical traps used to isolate particles as their motion is cooled to the quantum ground state, where excess photons result in additional heating and quantum decoherence (*82*), precision positioning of microscopic sensors (*83*), automated optical assembly of microscale structures (with the caveat that optimized trap shapes would need to be adapted if brought close to other particles) (*84*), and the study of photosensitive biological systems (*15*). More broadly, optimizing the spatial structure of optical tweezers could also be used to tune the interaction between multiple levitated particles, with potential applications to optical binding (*85*) and the synchronization of optomechanical oscillators (*86*). The work we have presented here strives for the optimal transfer of momentum from the photonic to the micro-mechanical regime, offering a route toward the fundamental limits of passive far-field optical control over matter.

## MATERIALS AND METHODS

### Simulated optimization of optical traps

Our modeling framework is built upon the freely available Optical Tweezers Toolbox (*47*), with several custom modifications to integrate the GWS operators that allow fast optical force and stiffness calculations. For the optimization itself, we used MATLAB’s “fmincon” function with the “interior-point” algorithm, which is designed for nonlinear constrained optimization. We note that the fmincon function is not capable of dealing with complex numbers, so we split ***u*** into its real and imaginary parts to perform the optimization.

We set κ*_x_* as the objective function to be maximized, and, then, to make sure that the solution light field has the desired stiffness and stability requirements, we set the following constraints. Mimicking the properties of the conventional optical trap, we want the transverse stiffness to be isotropic, so we require that κ*_y_* = κ_x_. In addition, to further cement this property and avoid solutions that preferentially treat the *y* = *x* or *y* = −*x* direction, we also require that κ_*y*=*x*,*y*=−*x*_ = κ*_x_*. For the *z* direction, we follow the symmetries of a diffraction-limited spot (i.e., lower stiffness axial trapping) and require that κ*_z_* = κ*_x_*/3.2. We do note, however, that any desired aspect ratio between the stiffnesses along different dimensions can be specified (see section S5). We further require that there is no optical force acting on the particle at the origin, *f*_*x*,*y*,*z*_(**0**) = 0, so as to ensure existence of a stable equilibrium. Last, we require normalized power such that ∣**u**∣^2^ = 1. We also note that some of these constraints can be removed if the basis in which **u** is expressed is itself limited to certain symmetries as, for example, is the case for our phase-only optimizer.

### Live experimental optimization

In our experimental optimization routine, we aim to minimize the number of optimization variables *N* to converge to a solution as rapidly as possible. We achieve this by exploiting knowledge of the symmetries of target particles and the optical system itself. We mimic the Bessel basis used in simulations by splitting our SLM screen into *N* evenly radially spaced rings and aim to determine the relative phase that should be imparted to light reflecting from each ring. In this geometry, use of circularly polarized light limits the search space to cylindrically symmetric fields, well matched to the spherically shaped target particles, as long as the optical system is well aligned and aberrations are minimized. For live optimization, we exclusively use light that does not carry OAM, and our simulations presented in section S31 indicate that there is nothing to be gained from introducing OAM.

The phase of each ring is optimized as follows. At the start of each iteration, the optimizer randomly selects half of the *N* rings and adds a small phase change Δϕ to them. The particle’s CoM is then tracked for a time Δ*t* to accumulate enough data for evaluating the trap stiffness **κ**_+Δϕ_ [using the Equipartition theorem; (*2*)]. Next, Δϕ is subtracted from the same set of rings, and **κ**_−Δϕ_ is evaluated. We then also perform the stiffness evaluation on the initial phase configuration; this way, we avoid the optimizer getting stuck in a noise-induced false “high-stiffness” configuration. From the three-phase configurations, the one with the best stiffness is selected; this completes one iteration, and the process is then repeated. The “best field” within each iteration is determined as the one that increases κ_*x*,*y*_ and does not decrease κ*_z_* (or vice versa); if none of the three fields satisfy this condition, then the optimizer retains the initial field. We rely on the symmetries of the rings, the spherical particle itself, and circularly polarized light to ensure that κ*_y_* does not diverge substantially from κ*_x_*.

Substantial measurement times Δ*t* are required for precise and accurate measurements of trap stiffness, due to the stochastic nature of Brownian motion (*87*). In order not to wait too long per iteration, we use the shortest time that still enables successful optimization, which means that the convergence process is inherently noisy as shown in Fig. 3C. The phase step size Δϕ tested at each iteration must be small enough to ensure that any trial fields projected onto the particle will not eject it from the trap and end the experiment. At the same time, Δϕ needs to be large enough so that the change in stiffness is detectable above the thermal noise, for the chosen measurement time Δ*t*. Typical values in our experiments were Δϕ = π/10 radians, *N* = 30, and Δ*t* = 10 s. We also avoid abrupt jumps in the phase profile displayed on the SLM (which lead to higher loss in diffraction efficiency) by linearly interpolating the phase between the *N* rings.

Our live-optimization algorithm is designed to be relatively tolerant of noisy experimental measurements (*70*). However, in comparison to the optimizer used to predesign optical traps, live optimization has a reduced search space, due to the need to maintain trap stability at all steps throughout the optimization pathway. This constraint limits the enhancements that live optimization can deliver when compared to the predesigned traps, even in noiseless conditions (see section S27).

### Holographic optical tweezers with 3D tracking

Our holographic optical tweezers setup is schematically detailed in Fig. 3A. It is based on a modified version of the “cube” optical tweezers platform presented in (*88*). A 1064-nm continuous-wave diode-pumped solid-state laser (Laser Quantum: VentusIR, 3) is expanded to fill a liquid crystal SLM (Boulder Nonlinear Systems: XY-series, 512 × 512 resolution), which is, in turn, reimaged onto the back of a 1.3 NA 100× oil-immersion objective (Olympus) using a 4*f*-imaging system. A sample slide holding a dilute suspension of silica microspheres (microParticles GmbH) is placed in the front focal plane of the objective, where the microspheres can be manipulated using wavefront-shaped optical traps.

We implement stereoscopic vision for 3D particle tracking. The sample is back-illuminated with two red LED sources, forming twin views of the sample from different angles. The two images are collected by the same objective lens and later passed through two spatially adjacent prisms, positioned in the Fourier plane of the sample, to separate the two “eyes” of the stereovision system. Last, the two spatially separated views of the sample are imaged side-by-side onto a high-speed camera (Mikrotron, EoSens CL). 2D center-of-symmetry–based real-time tracking in each image enables reconstruction of 3D microsphere trajectories using parallax (*33*), with nanometric axial precision (see section S25) (*54*). The system is operated using the LabVIEW-based “Red Tweezers” software (*89*), which is modified to incorporate stereovision 3D tracking. For this work, we made further changes to implement our custom live-optimization routine.

The optical traps used in the experiments presented here were generated using the first diffraction order from the light shaped by the SLM. We found that using the full NA of the objective lens resulted in higher levels of aberration. To combat this, we reduce the NA of our system from 1.3 to 1.13 by including a circular aperture on the phase masks displayed on the SLM; thus, only light reflecting from within the aperture is transmitted to the first diffraction order, cutting out light from the edges of the SLM. This circular aperture was used for all Gaussian and optimized trapping experiments to ensure an identical NA in all cases.

## Supplementary Materials

### This PDF file includes:

Sections S1 to S32 Figs. S1 to S33 Legend for movie S1
