# Supplementary material for: Photon-efficient optical tweezers via wavefront shaping
Source: Sci Adv. 2024 Jul 5;10(27):eadi7792. doi: 10.1126/sciadv.adi7792 (PMC11225778; doi:10.1126/sciadv.adi7792)
Supplement: Supplementary file 1 — Sections S1 to S32 Figs. S1 to S33 Legend for movie S1 [file sciadv.adi7792_sm.pdf]

Science Advances
AAAS

Supplementary Materials for

Photon-efficient optical tweezers via wavefront shaping

Unė G. Būtaitė *et al.*

Corresponding author: Unė G. Būtaitė, u.butaite@exeter.ac.uk;
Jonathan M. Taylor, jonathan.taylor@glasgow.ac.uk; David B. Phillips, d.phillips@exeter.ac.uk

Sci. Adv. **10**, eadi7792 (2024)
DOI: 10.1126/sciadv.adi7792

The PDF file includes:

Sections S1 to S32
Figs. S1 to S33
Legend for movie S1

Other Supplementary Material for this manuscript includes the following:

Movie S1

S1. 1D GLOBALLY-OPTIMAL FIELDS USING GWS OPERATORS

Eigenvalue-based approaches such as the Optical Eigenmodes framework [37] or the Generalised Wigner-Smith (GWS) operators [40] can be used to identify light fields which are globally-optimal in *one dimension* for a chosen aspect of light-particle interaction [38, 41]. We note that the GWS approach only requires far-field information, while the optical eigenmode method necessitates a relation between the far-field and the near-field around the target – access to which is typically not possible in an experiment. In this section we describe how 1D optimisation can be achieved in the GWS framework, and examine the shortcomings of optimising one dimension only.

The GWS operators are a broad category of operators which were obtained by generalising the well-established Wigner-Smith operator [50, 51]; they take the form of:

$$\boldsymbol{Q}_\alpha = -\mathrm{i}\boldsymbol{S}^{-1}\frac{\partial \boldsymbol{S}}{\partial \alpha}, \tag{S1}$$

where $\boldsymbol{S}$ is the scattering matrix such that $\boldsymbol{u}_{\text{out}} = \boldsymbol{S}\boldsymbol{u}$; here $\boldsymbol{u}$ is a column vector describing the incident field, and $\boldsymbol{u}_{\text{out}}$ is a column vector describing the outgoing field (i.e. after the incident field is scattered by the particle). The variable α with respect to which the scattering matrix is differentiated can be any global or local property of the system. For example, frequency of the incident light, or orientation, position, or size of a scatterer.

The remarkable property of these GWS operators is that the eigenvalues corresponding to the eigenstates of $\boldsymbol{Q}_\alpha$ are directly proportional to a variable conjugate to α. Frequency is paired with time, orientation with angular momentum, position with linear momentum (and by extension force), size with radiation pressure [41]. For example, if a light field incident on the particle is an eigenstate of $\boldsymbol{Q}_x$, then the force exerted on the particle is proportional to the eigenvalue corresponding to this eigenstate. The field with maximum force is therefore simply given by the eigenstate of $\boldsymbol{Q}_x$ with the largest eigenvalue [41]. This concept can be extended to define a stiffness operator $\boldsymbol{K}$ by noting that stiffness is given by the force gradient with respect to particle-trap displacement: $\boldsymbol{K}_x = -\frac{\partial \boldsymbol{Q}_x}{\partial x}$.

In Fig. S1(a,b) we present examples of globally optimum stiffness eigenstates for x and y directions, for a micro-sphere of 4 µm radius and refractive index of 1.44, immersed in water ($n = 1.326$), and illuminated from the negative z-direction with circularly polarised laser light of wavelength 1.064 µm, through a numerical aperture (NA) of 1.25. Notable features of the x-optimal and y-optimal traps, in this case, are that they also simultaneously enhance stiffness in the other in-plane orthogonal direction to some extent. However, these traps are not stable in the axial direction, i.e. the z-force is

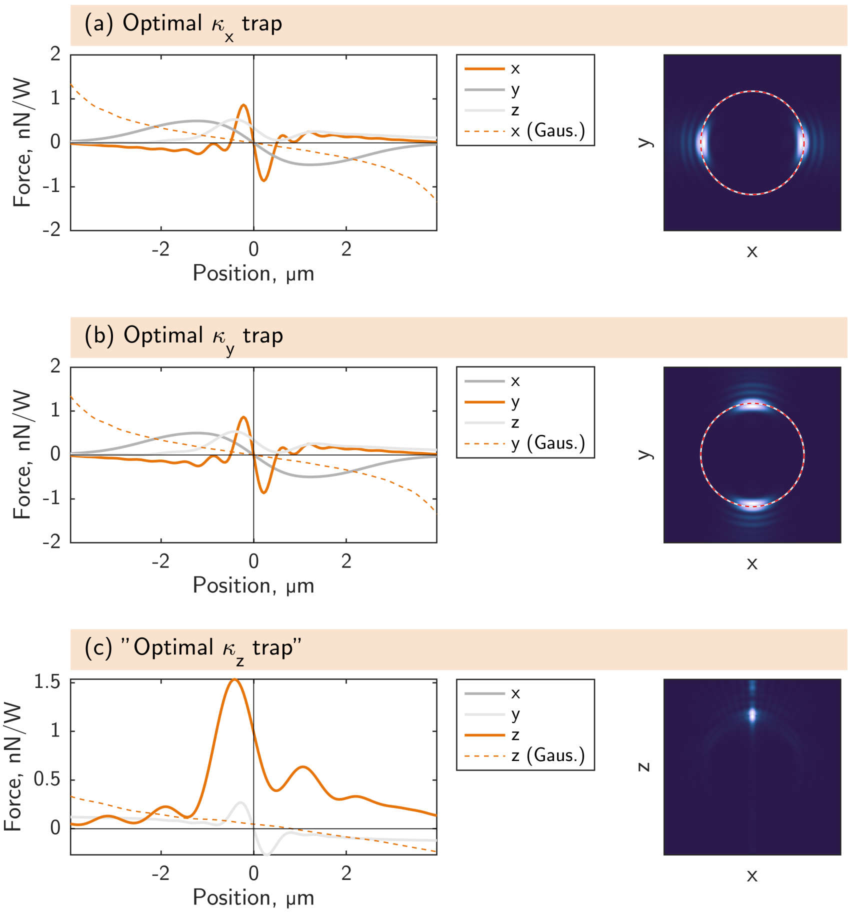

Figure S1: **1D globally optimal stiffness traps**.

always positive, pushing the particle out of the trap along the optical axis. As noted in the main paper, in general, 1D optimisation using the GWS operator places no constraints on the stiffness in other dimensions, or the axial stability of the particle. We also point out that the direction of beam propagation, z, is inherently different because of radiation pressure and scattering forces. So while the $\boldsymbol{K}_z$ operator can be used to identify the field with the highest z-force gradient, it does not actually make sense to refer to a 'stiffness' in this situation because the trap is inherently unstable along the same dimension (i.e. in z), as seen in Fig. S1(c).

Compared to a conventional optical trap the force response of optimised traps have sharper features (as is also seen in main Fig. 1B-D). Since the optimised traps direct most of the intensity to the edges of the trapped particle, particle displacements of several hundreds of nanometers can substantially change which part of the trap the particle is sampling, resulting in fluctuations in the force profile, as well as emergence of secondary equilibria. If the particle is optically trapped, however, it will not explore this region, and remain trapped within the linear force-response regime.

S2. CONFINEMENT VOLUME

The thermal motion of the centre-of-mass of a particle in an optical trap is characterised by a tri-variate Gaussian (normal) distribution. We define the confinement volume to be an ellipsoid, the principal radii of which are equal to three standard deviations of the particle's motion:

$$V_{\mathrm{c}} = \frac{4}{3}\pi(3\sigma_x)(3\sigma_y)(3\sigma_z), \tag{S2}$$

where, for example, σ_x is the standard deviation in the x-dimension.

In 1D, the probability of finding the particle within 3 standard deviations of the mean position is $p \sim 0.997$. Therefore, in 3D, the probability of finding the particle within 3 standard deviations of the mean, in any direction, is given by $p \sim 0.997^3 \sim 0.99$.

The standard deviation can be linked to stiffness via the Equipartition theorem, which equates the thermal energy stored in each degree of freedom, $\frac{1}{2}k_{\mathrm{B}}T$, to the optical potential energy, $\frac{1}{2}\kappa_x\sigma_x^2$, such that:

$$\sigma_x = \sqrt{\frac{k_{\mathrm{B}}T}{\kappa_x}}. \tag{S3}$$

Substituting this into Eq. S2 we get:

$$V_{\mathrm{c}} = 36\pi\sqrt{\frac{k_{\mathrm{B}}^3T^3}{\kappa_x\kappa_y\kappa_z}}. \tag{S4}$$

Note that in the notation we used here, x, y, z refer to the principal axes of the ellipsoid, not the Cartesian axes.

S3. $\boldsymbol{T}$-MATRIX FORMALISM

Any incident or outgoing light field ($\boldsymbol{E}_{\text{in}}$, $\boldsymbol{E}_{\text{out}}$ respectively) can be represented in the vector spherical harmonics (VSHs) basis:

$$\boldsymbol{E}_{\text{in}}(k\boldsymbol{r}) = \sum_{n=1}^{\infty} \sum_{m=-n}^{n} a_{nm}\boldsymbol{M}_{nm}^{(2)}(k\boldsymbol{r}) + b_{nm}\boldsymbol{N}_{nm}^{(2)}(k\boldsymbol{r}), \tag{S5}$$

$$\boldsymbol{E}_{\text{out}}(k\boldsymbol{r}) = \sum_{n=1}^{\infty} \sum_{m=-n}^{n} c_{nm}\boldsymbol{M}_{nm}^{(1)}(k\boldsymbol{r}) + d_{nm}\boldsymbol{N}_{nm}^{(1)}(k\boldsymbol{r}). \tag{S6}$$

Here $\boldsymbol{M}_{nm}^{1,2}, \boldsymbol{N}_{nm}^{1,2}$ are the VSHs as defined in the Optical Tweezers Toolbox (OTT) [47]; a, b, c, d are referred to as the beam shape coefficients (BSCs) and can be readily calculated using the OTT; k is the wavenumber of incident light and $\boldsymbol{r}$ is the spatial coordinate at which the field is evaluated. Note that VSHs are defined with respect to the location of the particle. The infinite sum is, in practice, cut off at a certain value n_{max}, which depends on the radius of the volume over which the field has to be accurately represented.

The BSCs describing the outgoing field c, d can be obtained by multiplying the BSCs of the incident field by the $\boldsymbol{T}$-matrix of the scattering particle (once again this matrix can be computed using OTT):

$$\begin{bmatrix} \boldsymbol{c} \\ \boldsymbol{d} \end{bmatrix} = \boldsymbol{T} \begin{bmatrix} \boldsymbol{a} \\ \boldsymbol{b} \end{bmatrix}, \tag{S7}$$

where $\boldsymbol{a}, \boldsymbol{b}, \boldsymbol{c}, \boldsymbol{d}$ are column vectors containing all the $a_{nm}, b_{nm}, c_{nm}, d_{nm}$ coefficients respectively.

S4. BESSEL BEAM BASIS

We chose to represent the incident field $\boldsymbol{u}$ in the Bessel beam basis since its symmetries are particularly well suited for light limited to the angle subtended by an objective lens, as is the case in optical tweezers. In the far-field (i.e. the entrance pupil of the objective) an ideal Bessel beam is an infinitesimally thin ring with a constant amplitude, and a phase varying according to $L\theta$, where L is the orbital angular momentum (OAM) of the beam and θ is the azimuthal angle. In our simulations we use experimentally more realistic non-ideal Bessel beams, which are rings of finite thickness. Each element in our incident field vector $\boldsymbol{u}$ represents the complex amplitude of one of these Bessel modes.

Since our modelling of light/particle interactions is conducted in the VSH basis, it is convenient to define a matrix $\boldsymbol{M}_{\text{b2v}}$ which transforms light fields expressed in the Bessel basis to light fields expressed in the VSH basis: $\boldsymbol{u}^{\text{VSH}} = \boldsymbol{M}_{\text{b2v}}\boldsymbol{u}$. Each column in $\boldsymbol{M}_{\text{b2v}}$ contains the BSCs $\boldsymbol{a}, \boldsymbol{b}$ for one Bessel mode (see S3); these coefficients are readily computed using OTT.

A typical input basis set in our simulations consists of 50 different radii rings (each with the same thickness), with OAM values up to ± 15. Note that we do not allow equally many OAM values for every ring – the OAM cutoff for smaller rings is lower, since the intensity distribution in the object plane of small radii and high OAM Bessels is such that most of the light lies well outside of the particle being trapped, and therefore do not affect it.

S5. CHANGING THE ASPECT RATIO OF THE TRAP STIFFNESS

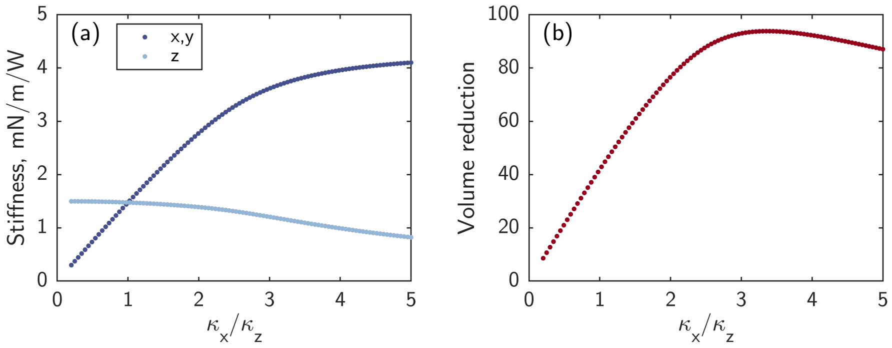

Figure S2: **Effect of desired aspect ratio of the trap on (a) stiffness and (b) volume reduction**. These results were simulated for a particle with a radius of 3.62 µm and a refractive index of 1.48, immersed in water ($n = 1.326$); the wavelength of light is 1.064 µm, and NA= 1.25.

Since there is no 'proper' definition of what an optimal trap looks like in 3D, it is up to the researcher to decide on the desired aspect ratio of the trap, i.e. the ratio between stiffnesses along different directions. To generate optical traps presented in the main paper we have chosen to be guided by the size of a diffraction limited spot. Starting with Abbe's resolution limit d in the transverse and axial directions:

$$d_{x,y} = \frac{\lambda}{2\mathrm{NA}}, \qquad d_z = \frac{2\lambda}{\mathrm{NA}^2}, \tag{S8}$$

we see that the desired ratio between transverse and axial stiffness is given by $\alpha = \kappa_x/\kappa_z = 4/\mathrm{NA}$, which is 3.2 for NA of 1.25 (here λ is the wavelength and NA is the numerical aperture of the objective). The transverse stiffness was chosen to be isotropic.

Figure S2 investigates how stiffness and volume reduction of the optimised trap change for different requested ratios κ_x/κ_z. We observe that at first the transverse stiffness increases linearly with increasing ratio and then starts attenuating, indicating that it becomes increasingly difficult for the optimiser to satisfy the z-stiffness constraint. We also see that there exists an optimal ratio (3.35 for the particle simulated here) at which the 3D volume reduction is maximised. This optimal ratio is dependent on particle parameters.

S6. SCATTERING MATRIX S AND ITS DIFFERENTIALS

The scattering matrix $\boldsymbol{S}$ is such that $\boldsymbol{u}_{\text{out}} = \boldsymbol{S}\boldsymbol{u}$. Here $\boldsymbol{u}$ is the incident light field expressed in the Bessel basis in the far-field; $\boldsymbol{u}_{\text{out}}$ is the outgoing field after it has been scattered by the particle, and is expressed in the VSH basis. Since the incoming and outgoing fields are represented in different bases, $\boldsymbol{S}$ has to bridge the two and is defined as:

$$\boldsymbol{S} = \boldsymbol{T}\boldsymbol{M}_{\text{b2v}}, \tag{S9}$$

where the $\boldsymbol{T}$-matrix describes scattering in the VSHs basis (as presented in S3) and $\boldsymbol{M}_{\text{b2v}}$ is the transform matrix from Bessel to VSHs basis (as defined in S4). In the following we derive a new analytical approach for calculating the 1st and 2nd differentials of $\boldsymbol{S}$, which are needed to calculate the GWS operators.

Let $\boldsymbol{T}(x)$ be a $\boldsymbol{T}$-matrix describing how a particle (located at a distance x from the origin) scatters the light field, and let $\boldsymbol{T}_0$ be the $\boldsymbol{T}$-matrix describing scattering by the particle when located at the origin $x = 0$. The two are related by:

$$\boldsymbol{T}(x) = \boldsymbol{A}(-x)\boldsymbol{T}_0\boldsymbol{A}(x), \tag{S10}$$

where $\boldsymbol{A}(x)$ is a translation matrix that translates the origin with respect to which the VSHs are defined to position x.

Our scattering matrix $\boldsymbol{S}(x)$ for a particle located at x is then given by:

$$\boldsymbol{S}(x) = \boldsymbol{T}(x)\boldsymbol{M}_{\text{b2v}} = \boldsymbol{A}(-x)\boldsymbol{T}_0\boldsymbol{A}(x)\boldsymbol{M}_{\text{b2v}}, \tag{S11}$$

and the $\boldsymbol{Q}$ and $\boldsymbol{K}$ operators are given by:

$$\boldsymbol{Q}_x = -\mathrm{i}\boldsymbol{S}^{-1}\frac{\mathrm{d}\boldsymbol{S}}{\mathrm{d}x} = -\mathrm{i}\boldsymbol{S}^{\dagger}\frac{\mathrm{d}\boldsymbol{S}}{\mathrm{d}x}, \tag{S12}$$

$$\boldsymbol{K}_x = -\frac{\mathrm{d}\boldsymbol{Q}_x}{\mathrm{d}x} = \mathrm{i}\left[\frac{\mathrm{d}\boldsymbol{S}^{-1}}{\mathrm{d}x}\frac{\mathrm{d}\boldsymbol{S}}{\mathrm{d}x} + \boldsymbol{S}^{-1}\frac{\mathrm{d}^2\boldsymbol{S}}{\mathrm{d}x^2}\right] = \mathrm{i}\left[\left(\frac{\mathrm{d}\boldsymbol{S}}{\mathrm{d}x}\right)^{\dagger}\frac{\mathrm{d}\boldsymbol{S}}{\mathrm{d}x} + \boldsymbol{S}^{\dagger}\frac{\mathrm{d}^2\boldsymbol{S}}{\mathrm{d}x^2}\right], \tag{S13}$$

where we have used the fact that the scattering matrix $\boldsymbol{S}$ is unitary ($\boldsymbol{S}^{-1} = \boldsymbol{S}^{\dagger}$, see S7).

The differentials of $\boldsymbol{S}$ could be evaluated numerically by changing the x-location of the particle several times, evaluating $\boldsymbol{S}$ at those locations, and then using finite difference schemes. This is, however, computationally expensive because of the need to repeatedly translate the origin with respect to which the VSHs are defined (i.e. calculate $\boldsymbol{A}(\pm x)$ and left- and right-multiply $\boldsymbol{T}_0$). We therefore develop analytical expressions for evaluating these derivatives.

The differentials we need are given by:

$$\frac{\mathrm{d}\boldsymbol{S}}{\mathrm{d}x} = \frac{\mathrm{d}\boldsymbol{T}}{\mathrm{d}x}\boldsymbol{M}_{\text{b2v}}, \qquad \frac{\mathrm{d}^2\boldsymbol{S}}{\mathrm{d}x^2} = \frac{\mathrm{d}^2\boldsymbol{T}}{\mathrm{d}x^2}\boldsymbol{M}_{\text{b2v}}, \tag{S14}$$

so we are really interested in finding the differentials of $\boldsymbol{T}$.

Since we are interested in small particle displacements only, we can Taylor-expand $\boldsymbol{T}(x)$ and $\boldsymbol{A}(x)$ about $x = 0$:

$$\boldsymbol{T}(x=0) = \boldsymbol{T}_0 + \frac{\mathrm{d}\boldsymbol{T}}{\mathrm{d}x}x + \frac{1}{2}\frac{\mathrm{d}^2\boldsymbol{T}}{\mathrm{d}x^2}x^2 + \mathcal{O}(x^3) \tag{S15}$$

$$\boldsymbol{A}(x=0) = \boldsymbol{I} + \frac{\mathrm{d}\boldsymbol{A}}{\mathrm{d}x}x + \frac{1}{2}\frac{\mathrm{d}^2\boldsymbol{A}}{\mathrm{d}x^2}x^2 + \mathcal{O}(x^3), \tag{S16}$$

where $\boldsymbol{I}$ is the identity matrix. If we take the Taylor expansion of the translation matrices $\boldsymbol{A}$ and substitute them into Eq. S10, we get (only including terms up to x^2):

$$\boldsymbol{T}(x) = \boldsymbol{T}_0 + \left[\boldsymbol{T}_0\frac{\mathrm{d}\boldsymbol{A}}{\mathrm{d}x} - \frac{\mathrm{d}\boldsymbol{A}}{\mathrm{d}x}\boldsymbol{T}_0\right]x + \left[-\frac{\mathrm{d}\boldsymbol{A}}{\mathrm{d}x}\boldsymbol{T}_0\frac{\mathrm{d}\boldsymbol{A}}{\mathrm{d}x} + \frac{1}{2}\left(\frac{\mathrm{d}^2\boldsymbol{A}}{\mathrm{d}x^2}\boldsymbol{T}_0 + \boldsymbol{T}_0\frac{\mathrm{d}^2\boldsymbol{A}}{\mathrm{d}x^2}\right)\right]x^2. \tag{S17}$$

If we compare the above equation to the Taylor expansion of the $\boldsymbol{T}$-matrix in Eq. S15, we can directly read off the expressions for $\frac{\mathrm{d}\boldsymbol{T}}{\mathrm{d}x}$ and $\frac{\mathrm{d}^2\boldsymbol{T}}{\mathrm{d}x^2}$ as the terms in the square brackets. These can then be used to calculate $\frac{\mathrm{d}\boldsymbol{S}}{\mathrm{d}x}$ and $\frac{\mathrm{d}^2\boldsymbol{S}}{\mathrm{d}x^2}$ using Eqs. S14.

We now, of course, need to know the derivatives of the translation matrices $\boldsymbol{A}$. In a practical numerical implementation, the elements of the translation matrices are computed using recurrence relations, starting from a seed that is proportional to $j_n(x)$ (a spherical Bessel function of the first kind). From the linear nature of the recurrence relations it follows that *all* elements are proportional to $j_n(x)$, with no other dependence on x. Therefore the derivative of the translation matrix can be computed simply by substituting the seed $j_n(x)$ with its derivative $\left.\frac{\mathrm{d}j_n}{\mathrm{d}x}\right|_{x=0}$. The relevant derivatives required to

compute $\frac{\mathrm{d}\boldsymbol{A}}{\mathrm{d}x}$ and $\frac{\mathrm{d}^2\boldsymbol{A}}{\mathrm{d}x^2}$ (evaluated at $x = 0$) are:

$$
\begin{aligned}
\frac{\mathrm{d}j_0}{\mathrm{d}x} &= 0 & \frac{\mathrm{d}^2 j_0}{\mathrm{d}x^2} &= -\frac{1}{3} \\
\frac{\mathrm{d}j_1}{\mathrm{d}x} &= \frac{1}{3} & \frac{\mathrm{d}^2 j_1}{\mathrm{d}x^2} &= 0 \\
\frac{\mathrm{d}j_2}{\mathrm{d}x} &= 0 & \frac{\mathrm{d}^2 j_2}{\mathrm{d}x^2} &= \frac{2}{15} \\
\frac{\mathrm{d}^2 j_n}{\mathrm{d}x^2} &= 0 & \frac{\mathrm{d}^2 j_n}{\mathrm{d}x^2} &= 0 \quad \text{for } n > 2.
\end{aligned}
$$

Thus we have the means to analytically calculate the derivatives of $\boldsymbol{S}$ and avoid the use of numerical finite difference schemes.

S7. UNITARITY OF $\boldsymbol{S}$

In calculating $\boldsymbol{Q}$ and $\boldsymbol{K}$ we have used the property that $\boldsymbol{S}^{-1} = \boldsymbol{S}^{\dagger}$, which only holds for unitary matrices. In the following we demonstrate that our scattering matrix $\boldsymbol{S}$ is indeed unitary.

We begin by splitting $\boldsymbol{S}$ into two parts:

$$\boldsymbol{S} = \boldsymbol{B} + \boldsymbol{P}, \tag{S18}$$

where $\boldsymbol{B}$ corresponds to the incident field before it interacts with the particle (in our case this field is made up from a number of different Bessel beams), and $\boldsymbol{P}$ corresponds to the scattered field.

If $\boldsymbol{S}$ was unitary, then it would satisfy $\boldsymbol{S}^{\dagger}\boldsymbol{S} = \boldsymbol{I}$. We have:

$$
\begin{aligned}
\boldsymbol{S}^{\dagger}\boldsymbol{S} &= \left(\boldsymbol{B}^{\dagger} + \boldsymbol{P}^{\dagger}\right)\left(\boldsymbol{B} + \boldsymbol{P}\right) && \text{(S19)} \\
&= \boldsymbol{B}^{\dagger}\boldsymbol{B} + \left[\boldsymbol{B}^{\dagger}\boldsymbol{P} + \boldsymbol{P}^{\dagger}\boldsymbol{B} + \boldsymbol{P}^{\dagger}\boldsymbol{P}\right]. && \text{(S20)}
\end{aligned}
$$

From theory we know that *infinite* Bessel beams are orthogonal, therefore, $B_{\infty}^{\dagger}B_{\infty} = \boldsymbol{I}$, where the subscript ∞ indicates that the matrix is infinite in one dimension, i.e. $n_{\max}$ is infinite (note also that $\boldsymbol{P}$ effectively terminates at $n_{\max}$ corresponding to the bead radius, because the BSCs of the scattered field for $n > n_{\max}$ are zero). So if the term in the square brackets is zero, then we also have $S_{\infty}^{\dagger}S_{\infty} = \boldsymbol{I}$. We have confirmed numerically that indeed $\boldsymbol{B}^{\dagger}\boldsymbol{P} + \boldsymbol{P}^{\dagger}\boldsymbol{B} + \boldsymbol{P}^{\dagger}\boldsymbol{P} = 0$; note that this is true for finite matrices too.

We have now satisfied ourselves that $\boldsymbol{S}_{\infty}$ is unitary, and its inverse is equal to its conjugate transpose. But in practical calculations we always use a truncated $\boldsymbol{S}$. Let's see how this works out.

The GWS operator is given by:

$$
\begin{aligned}
\boldsymbol{Q}_x &= -\mathrm{i}\boldsymbol{S}_{\infty}^{-1}\frac{\mathrm{d}\boldsymbol{S}_{\infty}}{\mathrm{d}x} && \text{(S21)} \\
&= -\mathrm{i}\boldsymbol{S}_{\infty}^{-1}\left(\frac{\mathrm{d}B_{\infty}}{\mathrm{d}x} + \frac{\mathrm{d}\boldsymbol{P}}{\mathrm{d}x}\right) && \text{(S22)} \\
&= -\mathrm{i}\boldsymbol{S}_{\infty}^{\dagger}\frac{\mathrm{d}\boldsymbol{P}}{\mathrm{d}x}, && \text{(S23)}
\end{aligned}
$$

where we have used $\frac{\mathrm{d}B_{\infty}}{\mathrm{d}x} = 0$, since the incident field is independent of the bead's location x. Since $\boldsymbol{P}$ effectively terminates at $n_{\max}$, there is no need for an infinite S_{∞} because its non-zero terms beyond $n_{\max}$ are multiplied by the zeros in $\boldsymbol{P}$. It is therefore perfectly fine to use a truncated version of $\boldsymbol{S}$.

S8. GRADIENTS AND HESSIANS IN THE OPTIMISER

The optimisation algorithm that we employed in this work (MATLAB's *fmincon* with interior-point algorithm) benefits highly from having access to analytical expressions for the gradients and Hessians of the objective and constraint functions (with respect to the optimisation variables), otherwise they would have to be evaluated numerically at great computational expense. In our case, the objective function is given by $\kappa_x = \boldsymbol{u}^\dagger \boldsymbol{K}_x \boldsymbol{u}$. Differentiating this with respect to $\boldsymbol{u}$ we get the gradient $\nabla_{\boldsymbol{u}} \kappa_x$ and the Hessian $\mathrm{H}_{\boldsymbol{u}} \kappa_x$:

$$\nabla_{\boldsymbol{u}} \kappa_x = 2 \frac{\mathrm{d}\kappa_x}{\mathrm{d}\boldsymbol{u}^\dagger} = 2\boldsymbol{K}_x \boldsymbol{u}, \qquad \mathrm{H}_{\boldsymbol{u}} \kappa_x = \frac{\mathrm{d}^2 \boldsymbol{K}_x}{\mathrm{d}\boldsymbol{u}\mathrm{d}\boldsymbol{u}^\dagger} = 2\boldsymbol{K}_x. \tag{S24}$$

However, since the *fmincon* function does not work directly with complex numbers, we have to split the optimisation variables into their real and imaginary parts, such that $\boldsymbol{u} = \boldsymbol{a} + \mathrm{i}\boldsymbol{b}$. If we then define a real column vector $\boldsymbol{x} = [\boldsymbol{a}; \boldsymbol{b}]$, we can express κ_x as:

$$\kappa_x = \boldsymbol{x}^\mathrm{T} \boldsymbol{D}_x \boldsymbol{x}, \qquad \text{with } \boldsymbol{D}_x = \begin{bmatrix} \boldsymbol{K}_x & \mathrm{i}\boldsymbol{K}_x \\ -\mathrm{i}\boldsymbol{K}_x & \boldsymbol{K}_x \end{bmatrix}. \tag{S25}$$

In which case the gradient and Hessian are:

$$\nabla_{\boldsymbol{x}} \kappa_x = (\boldsymbol{D}_x + \boldsymbol{D}_x^\mathrm{T})\boldsymbol{x}, \tag{S26}$$

$$\mathrm{H}_{\boldsymbol{x}} \kappa_x = \boldsymbol{D}_x + \boldsymbol{D}_x^\mathrm{T}. \tag{S27}$$

The derivatives for the constraint functions are derived in the same fashion. For example, one of the constraints in our optimiser is that stiffness in the y-direction must be the same as stiffness in the x-direction. The corresponding constraint function is $g(\boldsymbol{x}) = \kappa_x - \kappa_y = 0$, and it can be differentiated as above. Equivalent expressions can be derived for the optical forces f^{opt} as well.

S9. OPTIMISER CONVERGENCE TIME

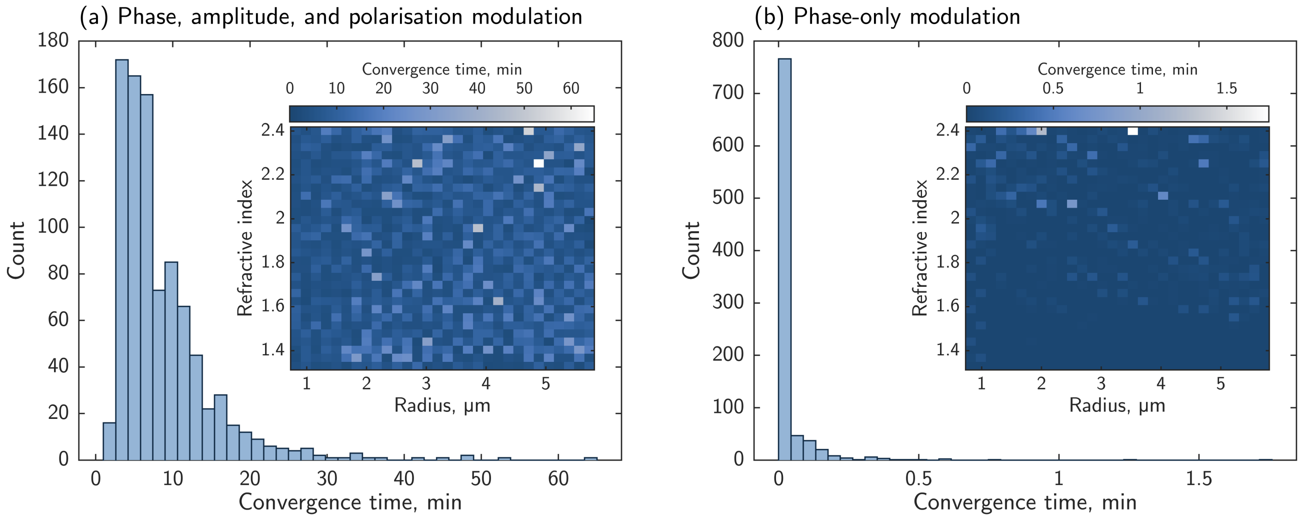

Figure S3: **Convergence time of the optimiser**. (a) If complex amplitude modulation is used, (b) if phase-only modulation is used.

Here we present an analysis of our optimiser convergence time for the 900 particle cases that we simulated. In the case of phase, amplitude, and polarisation modulation with the large 820-mode basis, 95% of cases took under 20 min to converge to a solution, and with the 50-mode phase-only modulation basis 99% of cases took less than 0.5 min, with one outlier that took just under 1.7 min. The insets in Fig. S3 show the convergence time for each of the 900 particles that we simulated – here we do not observe any clear trends based on particle size or refractive index. We also note that these values do not include the one-time calculation of GWS operators – this computation takes ~ 0.2–32 min depending on particle size (since larger particles require a higher number of terms in the VSH expansion, see S3).

These simulations were performed on an AMD Ryzen 9 3900X 12-Core Processor with 64GB RAM. Times quoted above for individual optimisations are for computations run on a single core. All 12 cores were used in parallel to reduce the overall time needed to simulate 900 particles.

S10. FORCE LANDSCAPE

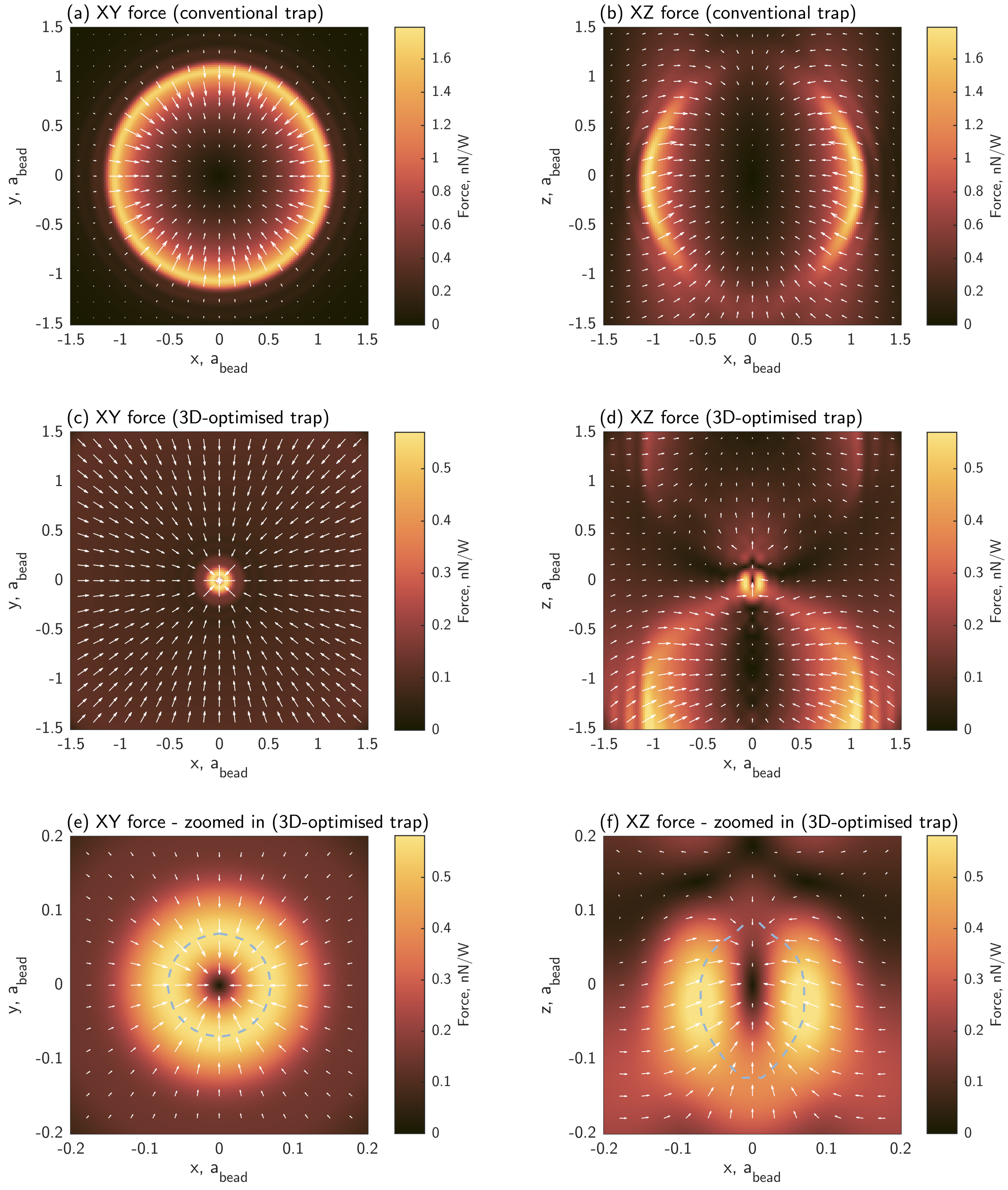

Figure S4: **Force landscape cross-sections of the conventional (a-b) and 3D-optimised (c-f) traps presented in Fig. 1**. The dashed blue contours in (e-f) indicate the trap capture range where the gradient of the restoring force (i.e. the stiffness) has been enhanced by the optimisation.

S11. FAR-FIELD SPATIAL PROFILE

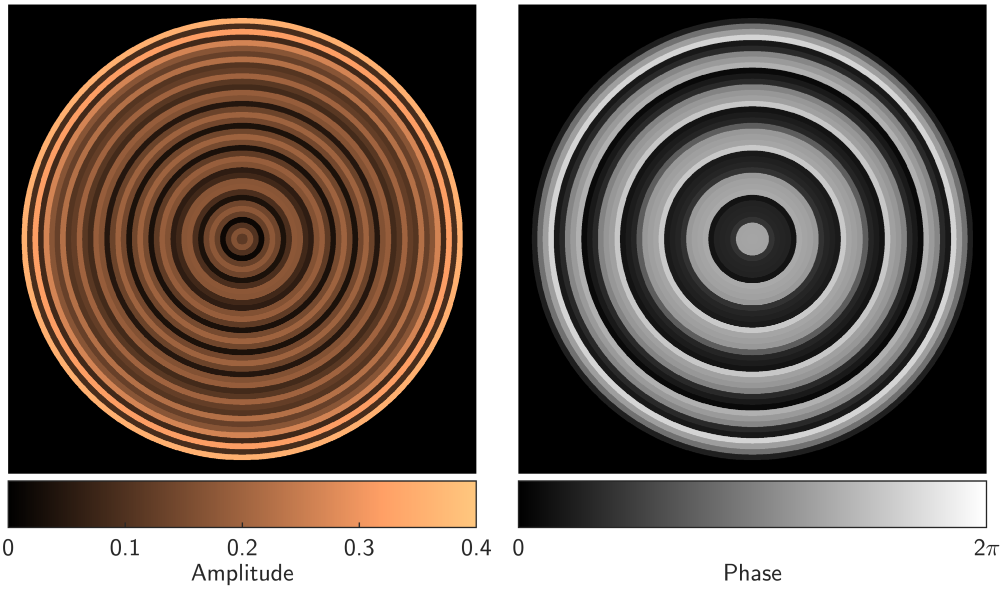

Figure S5: **Far-field profile of the simulated trap presented in Fig. 1**

S12. POWER REDUCTION FACTOR

By noting that stiffness is directly proportional to the power P of the trapping beam [2], we can obtain an expression for improvement in trap efficiency η^{rel} in place of confinement volume reduction $V_{\mathrm{c}}^{\mathrm{rel}}$. Say that the optical power of a trap is increased by η^{rel}, so that the stiffness also increases η^{rel} times in every direction. The confinement volume (as defined in Eq. 1 in the main text) is now accordingly:

$$V_{\mathrm{c}}^{\mathrm{low}\,P} \propto \frac{1}{\sqrt{\kappa_x \kappa_y \kappa_z}}, \qquad V_{\mathrm{c}}^{\mathrm{high}\,P} \propto \frac{1}{\sqrt{(\eta^{\mathrm{rel}})^3 \kappa_x \kappa_y \kappa_z}}. \tag{S28}$$

The volume reduction factor is then given by:

$$V_{\mathrm{c}}^{\mathrm{rel}} = \frac{V^{\mathrm{low}\,P}}{V^{\mathrm{high}\,P}} = \sqrt{\left(\eta^{\mathrm{rel}}\right)^3}. \tag{S29}$$

And it follows that:

$$\eta^{\mathrm{rel}} = \left(V_{\mathrm{c}}^{\mathrm{rel}}\right)^{2/3}. \tag{S30}$$

S13. DIFFERENT STARTING POINTS

Since the optimiser is not guaranteed to find the global optimum, the solution it finds can depend on the starting configuration. The results in the main paper use a conventional trap as a starting field for the optimiser. Here we investigate whether different starting fields result in different solutions, by testing 300 random starting fields for several different particles. Presented in the figure below are the x, y and z stiffnesses of the solution field for every starting point, for 5 beads with different radii a and refractive indices n. The first data point for every bead corresponds to the conventional trap starting field and matches the results presented in the main paper. We have observed no dependence between the solution and the starting beam, with the exception of one particle ($a = 4.45\,\mu\text{m}$, $n = 1.40$) where the solution alternates between two values (different by 0.01%). We therefore conclude that for the range of particles studied, the optimisation is robust to the starting field.

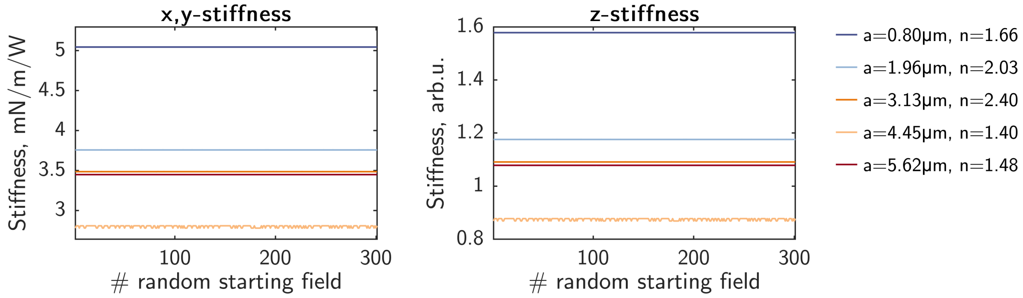

Figure S6: **Starting the optimiser with random fields.** Stiffness of the optimiser solution when starting it from different random initial field configurations. The first data point in every row corresponds to starting with a conventional tweezers beam. The optimisation was carried out with phase, amplitude, and polarisation modulation.

S14. STIFFNESS IN CONVENTIONAL AND 3D-OPTIMISED TRAPS

The conventional Gaussian profile optical tweezers, while simple to implement and versatile, do not perform equally well for different particles. For example, the stiffness goes down with particle size. Our 3D-optimised traps are individually shaped for every micro-sphere, and offer a more consistent performance across a range of sizes and refractive indices. Note also, that the ratio between transverse and axial stiffness is fixed in our optimiser (see S5), which is not the case for the conventional tweezers - here the particle properties determine the ratio.

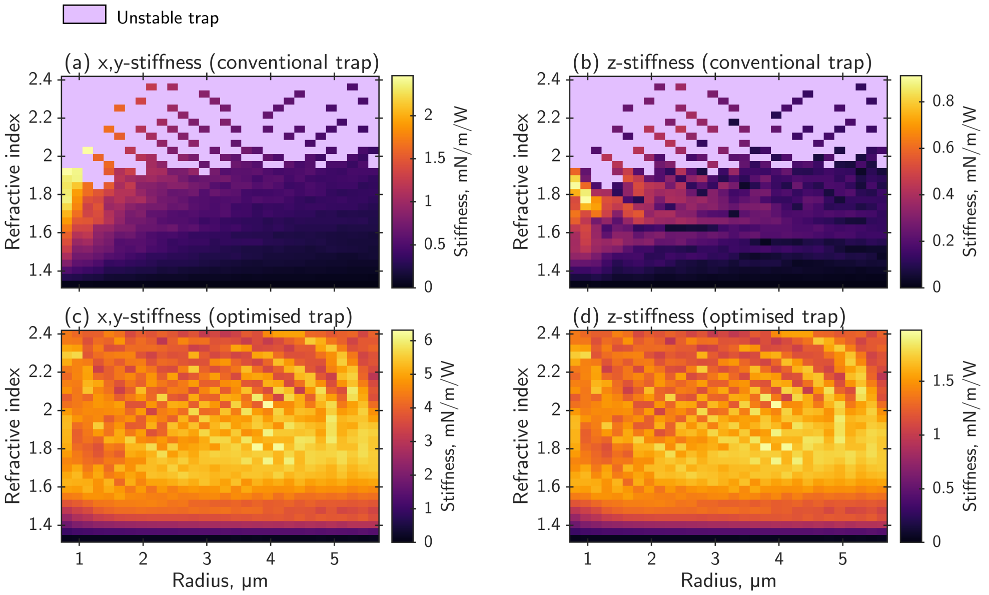

Figure S7: **Optical trap stiffness in a conventional (a-b) and 3D-optimised (c-d) traps**.

S15. ADDITIONAL INFORMATION FOR FIG.2G-H

In this section we present additional figures to aid visualisation of the 3D-optimised traps shown in Fig. 2G-H in the main text. Figure S8 shows the same data as Fig. 2G-H(left), but on a linear scale, instead of a log scale. We also present the fields at multiple cross-sections through the particles; depicting intensity in Figure S9 and amplitude (for visual clarity) in Fig. S10.

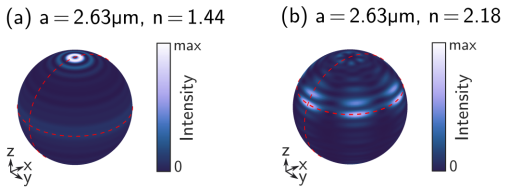

Figure S8: **Intensity on the particle surface**. Presented here are the same traps as shown in Fig. 2G-H, but plotted on a linear scale.

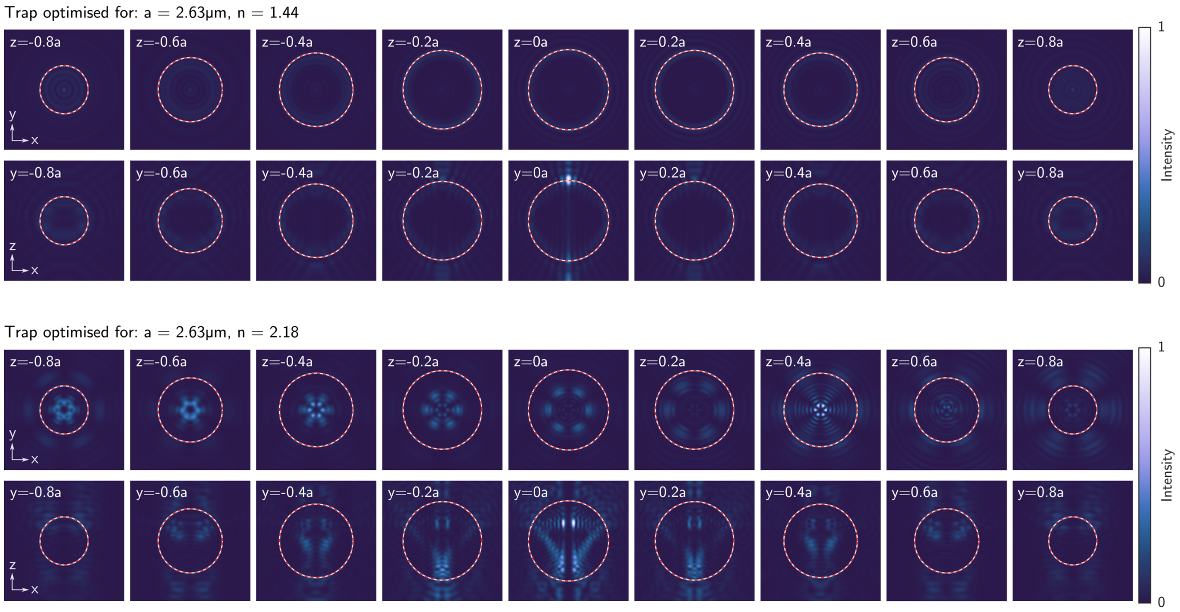

Figure S9: **Intensity at multiple cross-sections through the particle (same traps as shown in Fig. 2G-H)**. See also Fig. S10 below which shows the amplitude of these traps and better highlights the field shape near the edges of the particle.

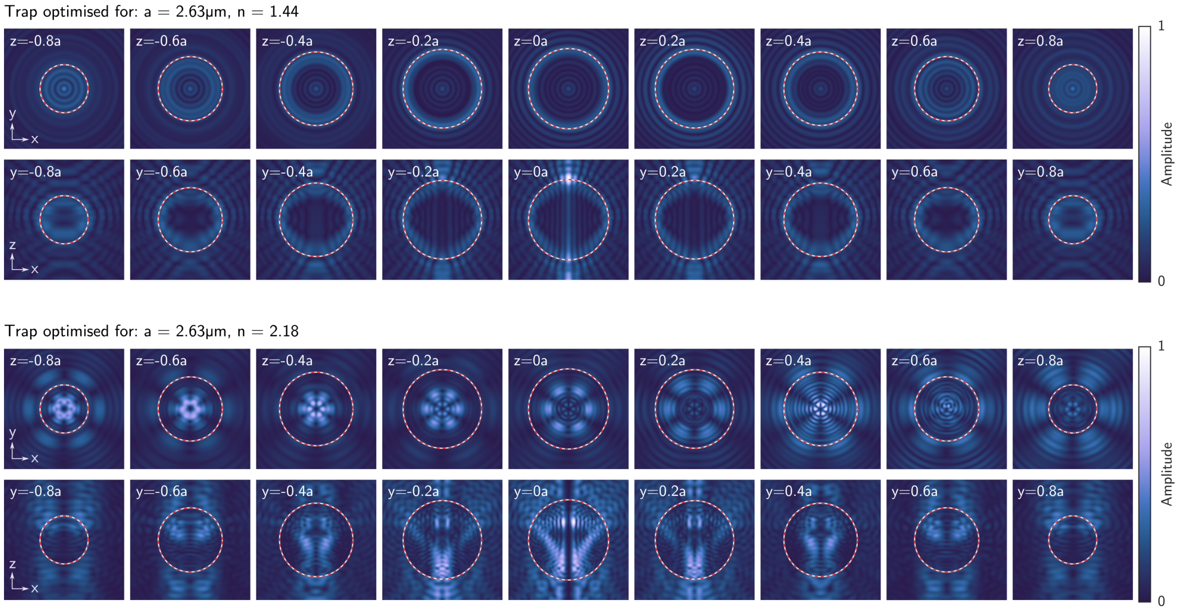

Figure S10: **Amplitude at multiple cross-sections through the particle (same traps as shown in Fig. 2G-H)**. Note that these are the same fields as in Fig. S9, but here amplitude instead of intensity is depicted.

S16. POLARISATION IN THE TRAPS PRESENTED IN FIG.2G-H

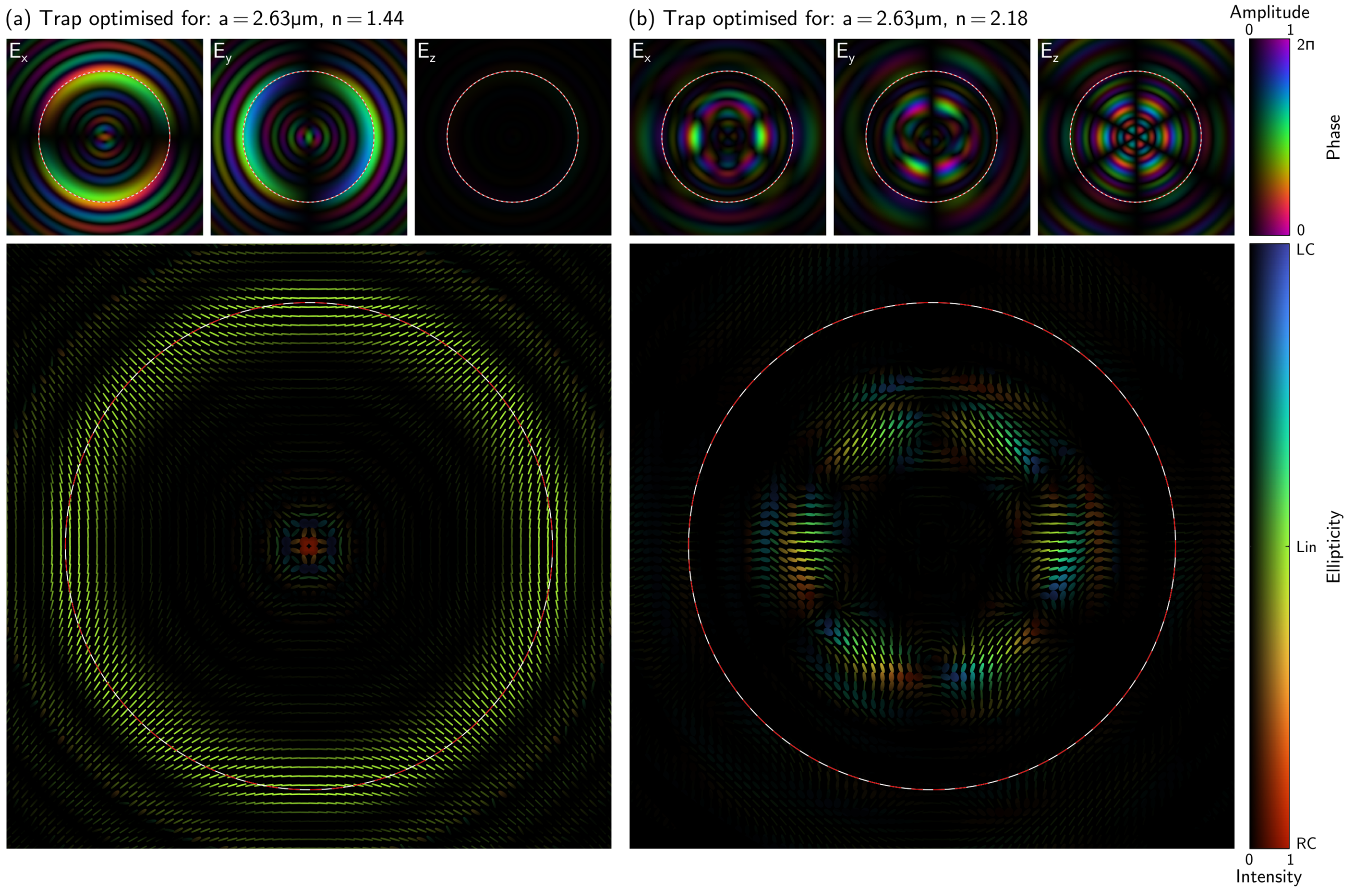

Figure S11: **Polarisation in the optimised traps for the two traps shown in Fig. 2G-H**. The top row shows the x, y, z components of the electric field E (at xy cross-sections, at $z = 0$). Here the colour represents the phase and brightness indicates the amplitude (the amplitude scaling is the same for all three components). The large plots below show the polarisation distribution in the traps. Here colour represents the ellipticity (LC - left circular, RC - right circular, Lin - linear) and brightness indicates the intensity; these plots were obtained using only the E_x and E_y components of the field. The white-red dashed line indicates the edges of the particle.

S17. PHASE-ONLY OPTIMISER

Given that full complex amplitude modulation is often not available in experimental setups, we also consider the phase-only light modulation scenario. We can see how the relevant equations will be transformed by splitting the complex amplitude $\boldsymbol{u}$ into its magnitude $\boldsymbol{a}$ and phase $\boldsymbol{\phi}$. The optical force is then given by:

$$f_x = \boldsymbol{u}^\dagger \boldsymbol{Q}_x \boldsymbol{u} \tag{S31}$$

$$= \left(\boldsymbol{a}^{\mathrm{T}} \circ \mathrm{e}^{-\mathrm{i}\boldsymbol{\phi}^{\mathrm{T}}}\right) \boldsymbol{Q}_x \left(\boldsymbol{a} \circ \mathrm{e}^{\mathrm{i}\boldsymbol{\phi}}\right), \tag{S32}$$

and likewise for κ_x. Here $\boldsymbol{a}$ and $\boldsymbol{\phi}$ are column vectors, superscript ^{T} indicates a transpose, and $\circ$ denotes a Hadamard (element-wise) product. Since the amplitude $\boldsymbol{a}$ is constant it can be absorbed into the $\boldsymbol{Q}_x$ operator, leaving phase $\boldsymbol{\phi}$ as the optimisation variable.

We make sure that the amplitude distribution in the far-field is uniform by limiting the input basis to only contain Bessel modes with 0-OAM. Thus, the modes constitute concentric annuli at the pupil plane of the objective lens. We also stipulate circularly polarised light at the pupil plane. Therefore, in addition to constraining the search space to phase-only light modulation in the pupil plane, here the fields are also constrained to possess circular symmetry – which is compatible with 3D trapping of micro-spheres. We note this constraint could be removed by representing the fields at the pupil in an alternative basis, such as a truncated plane wave basis or similar.

S18. COMPARISON OF DIFFERENT MODULATION TYPES

Reducing the number of parameters that can be optimised naturally affects the optimiser performance. In Fig. S12(a) we see that the achieved confinement volume increases by 1.1-9.5 times if polarisation is fixed (to circular). If modulation is constrained to phase-only (circular polarisation and uniform amplitude), the confinement volume is further increased by 1.1-9.0 times. Higher refractive index particles seem to benefit more from availability of amplitude and polarisation control. Full heat-maps showing stiffness enhancements, volume and power reduction, and stiffness distribution are presented in Figs. S13-S14.

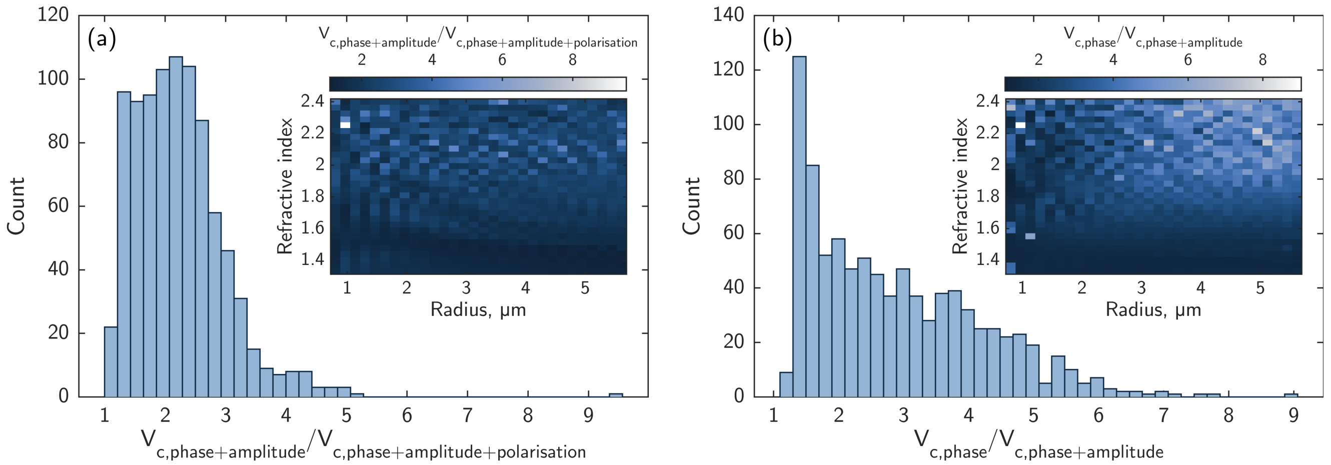

Figure S12: **Comparison of different modulation types.** (a) Ratio between x-stiffness κ_x of 3D-optimised optical traps created with and without polarisation modulation. (b) Ratio between x-stiffness κ_x of 3D-optimised optical traps created with and without amplitude modulation. Note, that because the ratio of stiffnesses is fixed in the optimiser, the ratios plotted in this figure would be identical for y and z stiffness.

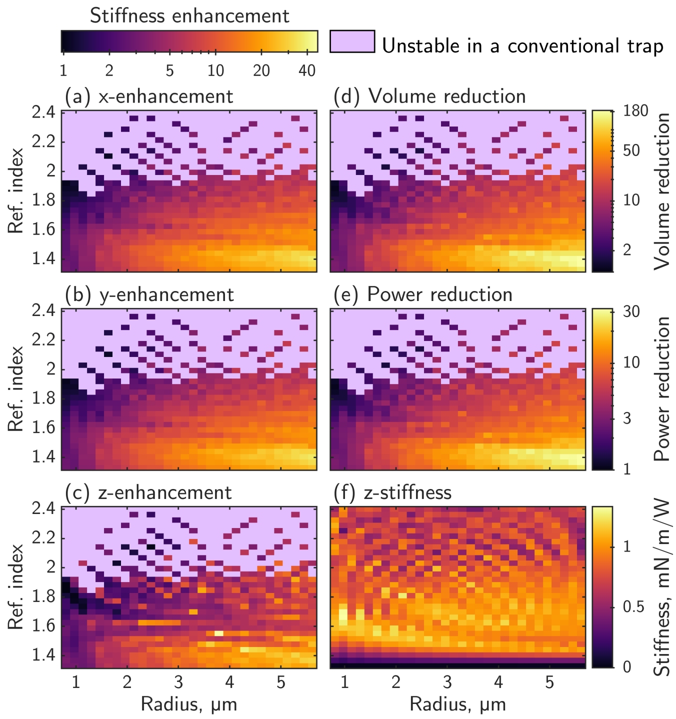

Figure S13: **Performance of 3D-optimised traps when using amplitude and phase modulation (polarisation is circular).**

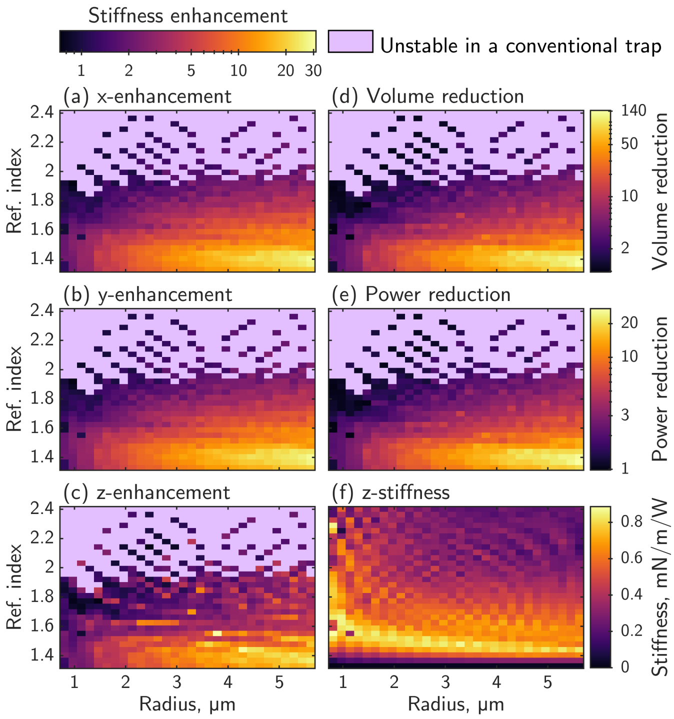

Figure S14: **Performance of 3D-optimised traps when using only phase modulation (polarisation is circular, amplitude is uniform).**

S19. POLARISATION EFFECTS

Linear polarisation breaks the focusing symmetry in high NA systems, and the optimiser has to adapt the solution field accordingly. Somewhat surprisingly, this does not decrease the performance. In fact, for a substantial portion of particle parameters confinement volume is smaller in linearly polarised traps, particularly for high refractive index particles, as seen in Fig. S15. We believe the reason for this is as follows. In the case of circular polarisation, the optimiser almost always defaults to using only one OAM value from all the available Bessel modes. This is an easy way to constrain the field to circular symmetry (in the transverse plane) and satisfy the $\kappa_y = \kappa_x$ requirement. But it also quite possibly prevents the optimiser from exploring further and making use of all the modes (as is the case with y-polarisation) to increase the stiffness further. Starting with random fields (as opposed to 0-OAM only Gaussian profile) did not affect this behaviour.

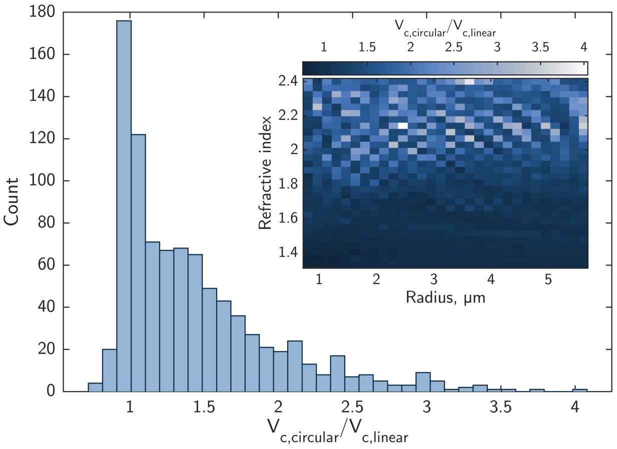

Figure S15: **Ratio between x-stiffness κ_x of linearly (along y) and circularly polarised 3D-optimised traps.**

Figure S16(a-f) shows the stiffness enhancements, volume and power reduction, and the stiffness distribution. Resulting solutions do not have circular symmetry, as seen in several examples in Figure S16(g-l). Instead, the most common trap formation consists of several spots of light in the transverse cross-sections.

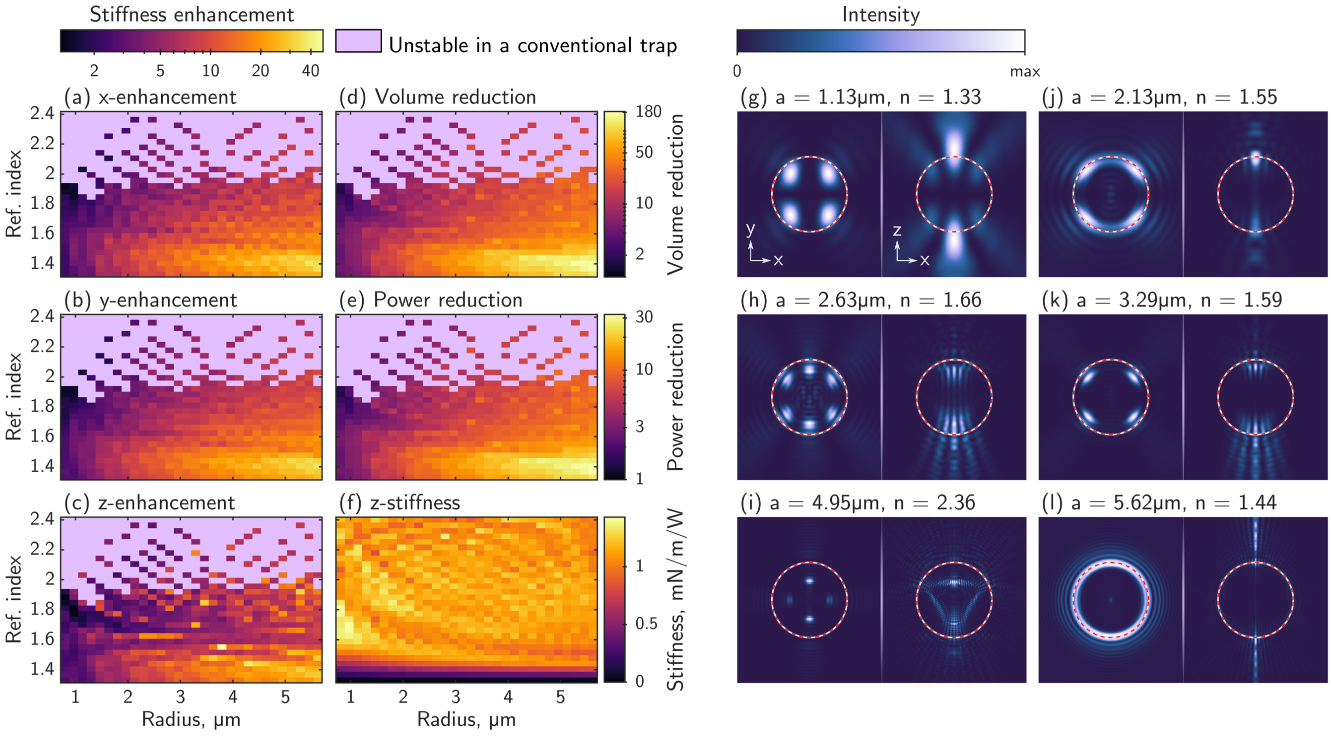

Figure S16: **3D-optimised trapping using linearly (along y) polarised light.**

S20. EFFECT OF PERTURBATIONS ON PRE-DESIGNED TRAP PERFORMANCE

Since our pre-designed optical traps are optimised for a very specific set of particle and environment properties, their performance rapidly degrades if they are employed in conditions other than they were designed for. One important factor is particle size. In Fig. S17 we simulate how the stiffness of a trap designed for a 5 µm radius micro-sphere with a refractive index of 1.45 changes if the particle has a slightly different radius. Interestingly, we observe that for small positive offsets in radius z-stiffness improves – but this is counteracted by a decrease in x-stiffness (and vice versa for small negative offsets). As the miss-match between the design and actual radius increases, stiffness in both directions starts to decrease. The sharp change in the trend at the offset of about -2.5% is a result of a large transition of the z-equilibrium location.

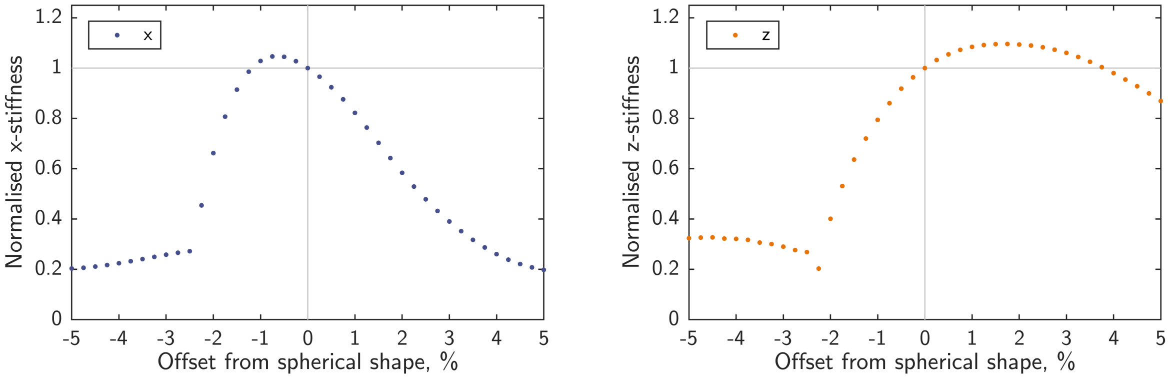

Figure S17: **Effect of bead size on pre-designed trap stiffness.**

Minor optical aberrations can have a profound effect on the fidelity with which pre-designed traps can be created in experiment. In Fig. S18 we numerically explore the effect of spherical aberration. The phase profile ζ of the aberration is described by:

$$\zeta = \alpha R_4^0, \tag{S33}$$

where R_4^0 is the radial Zernike polynomial associated with spherical aberration, and α is the strength (magnitude) of the aberration, i.e. $\alpha = 0$ constitutes no aberration, and other positive or negative values of α represent aberrations that increase with $|\alpha|$. To simulate the effect of spherical aberration, we add this aberrating phase profile to the far-field phase profile of the optimised trap, and observe how the trap performance changes with varying values of α. In Fig. S18 we see that the trap stiffness decreases rapidly upon the introduction of relatively small levels of spherical aberration. We note that here we have only considered spherical aberration, while real-world optical systems will likely also exhibit residual non-symmetric aberrations that will further contribute to a degradation in the trapping performance. The sensitivity of pre-designed optimsed traps to both particle geometry and minor system aberrations renders them challenging to be experimentally implemented – hence our use of live optimisation in this work.

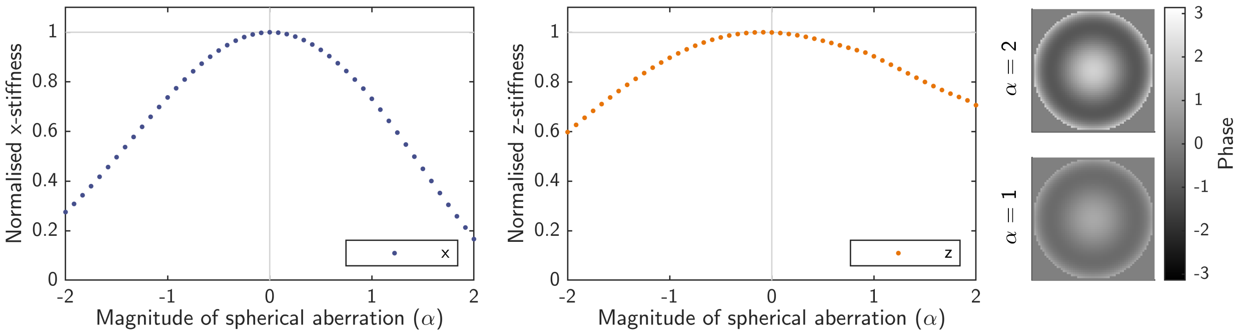

Figure S18: **Effect of spherical aberration on pre-designed trap stiffness in the x- (left) and z-directions (middle).** Simulated for a bead of radius 5 µm and refractive index 1.45. Two examples of the phase aberrations, ζ, in the pupil plane are shown in the right-most subplots.

S21. PRE-DESIGNED TRAPS IN AN EXPERIMENT

To try and remedy the imprecise knowledge of the vectorial optical transfer function of our high NA optical tweezers system, as well as of the precise sizes of the target micro-spheres, we have taken the following approach. We have pre-designed traps for seven different particle sizes within the standard deviation quoted by the manufacturer. We then tested each of these traps by incrementally rescaling the phase pattern in the pupil plane (by a total of 20% in steps of 1%) in an attempt to fine tune the intensity distribution (a strategy similar to that used in [27]).

Somewhat disappointingly, we did not observe enhancements matching the values predicted in the simulations. The best experimental volume reduction we achieved when applying a pre-designed trap in an experiment was 3.88, with corresponding stiffness enhancements of 3.80, 3.77, 1.05 in x, y, z respectively, for a 5 µm radius silica particle, when using a trap designed for 4.84 µm radius, after rescaling the trap to 80% of the SLM aperture. The expected volume reduction value for this trap was 36.00 (for a bead of 4.84 µm radius) with corresponding stiffness enhancements of 11.97, 11.97, 9.05 in x, y, z respectively. Figure S19 shows the particle trace for the conventional and pre-designed traps, illustrating the stiffness enhancement (or lack of it, in the case of the z-coordinate).

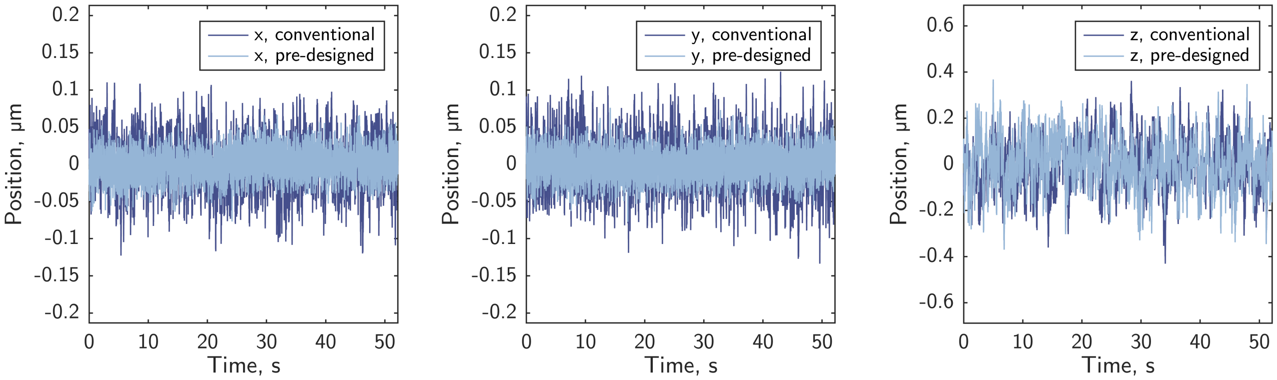

Figure S19: **Trace of a 5 µm radius silica micro-sphere when trapped in a conventional or a pre-designed trap**.

S22. SHAPE OF EXPERIMENTALLY OPTIMISED TRAPS

Since the live optimiser employs different methodology and yields smaller improvements than the pre-designed theoretical optimiser both in simulation and experiment, it is interesting to compare the actual shapes of the resulting traps. To recover the intensity distribution of our experimentally live-optimised traps, we place a reflective surface in front of the objective lens in our tweezers system and take images of the reflected laser beam - two examples of traps optimised for different size silica beads are shown in the right-most column of Fig. S20. We note that it is difficult to determine at which plane the bead would be stably trapped, so we image the plane at which the zero-order beam is in focus. Here we see the expected formation of rings, although for the smaller particle they do not extend all the way to the edges of the bead as they do in the pre-designed traps, and for the larger particle we see some intensity rings "overshoot" the particle. We do note, however, that reflection images are not an entirely faithful representation of the traps because they do not take into account scattering by the trapped particle or light field deformations when the trap is deployed deep in the sample beyond an oil-glass-water interface; nor can we image the axial cross-section in our system. We also note that these images were taken in response to reviewer comments many months after our main experiments were completed during which the optical system remained dormant. As such, aberrations visible in these images are not representative of the aberrations present during our experiments. For a more accurate representation please see inset in Fig. 3A.

Therefore, to get a better understanding of what to expect from the live-optimisation method we simulate it - the middle column in Fig. S20 shows examples of resulting traps. We see the familiar formation of rings in the transverse plane, however the central spot seems to be more prominent than in the pre-designed traps. The axial cross-sections further confirm that a lot of the intensity stays close to the central axis of the beads and remains "unused" resulting in lower stiffness enhancements.

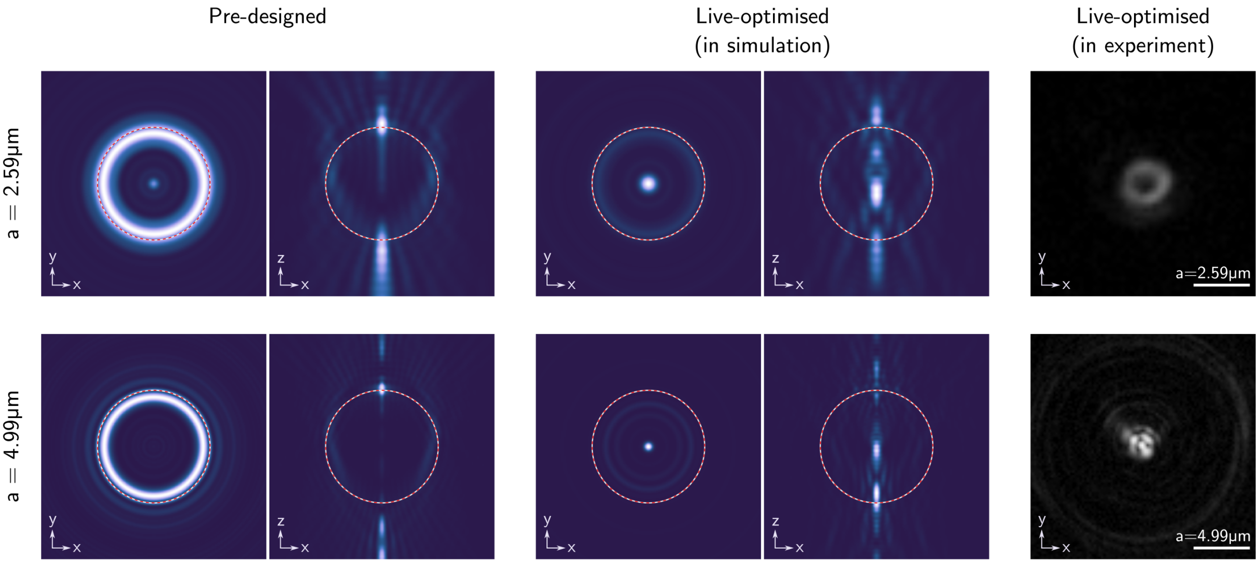

Figure S20: **Comparison of trap shapes obtained with different optimisers, for two silica micro-spheres with different radii.** The left column shows xy and xz cross-sections of the pre-designed optical traps, the middle column shows xy and xz cross-sections of traps obtained when simulating the live optimiser, and the right column shows reflection images of traps obtained when implementing the live optimiser in an experiment. All three optimisations used phase-only modulation in the far-field and circular polarisation. Note that we have moderately increased the contrast in the experimentally taken image for the bigger particle to make the outer rings more visible.

We also emphasise that the trap for the smaller particle looks similar to an OAM beam but this is not the case - our live optimiser does not employ OAM beams. It is also worth mentioning that light shaping visible in the reflection images clearly indicates that the live optimiser is different from aberration correction schemes, which merely improve on the "neatness" of the conventional Gaussian profile trap. For reference, in Fig. S21 we provide an image of the conventional optical trap, which is the starting point in our live optimiser.

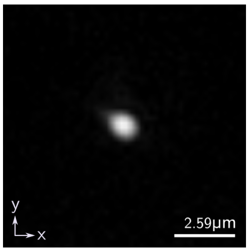

Figure S21: **Reflection image of a conventional trap in our optical tweezers system.**

S23. ADDITIONAL EXPERIMENTAL DATA

We experimentally optimised optical traps for silica micro-spheres with radii of 2.57, 4.01, 4.55, and 4.99 µm. To demonstrate the robustness of our live optimiser, we repeated the optimisation several times for the smallest and largest particles (each time optimising for a different particle). The full data set can be seen in Fig. S22. The trapped micro-spheres were displaced ∼25 µm from the bottom of the sample to ensure that the particle's thermal motion is not obstructed by any boundaries.

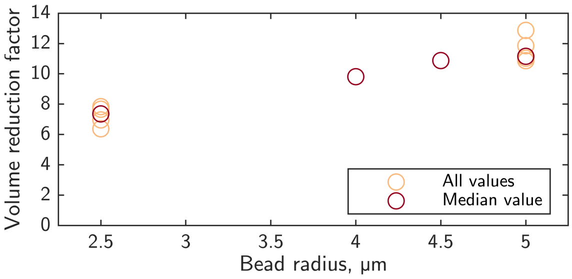

Figure S22: **Experimental volume reduction achieved with our live optimiser for different size micro-spheres**.

We also present several examples of the convergence behaviour of our live optimiser in Fig. S23. Here we observe different convergence rates and variations in the relationship between the transverse (x, y) and axial (z) directions. When performing these live optimisation experiments, care must be taken to make sure that the micro-sphere being trapped does not encounter any other particles, otherwise the tracking and stability of the trap might be compromised. To prevent this, it was sometimes necessary to manually translate the microscope stage, causing some the sudden sharp deviations in some of the plots in Fig. S23. External disturbances to the optical setup also result in similar deviations. It can be seen that the optimiser recovers rapidly and can continue to improve the trapping stiffness.

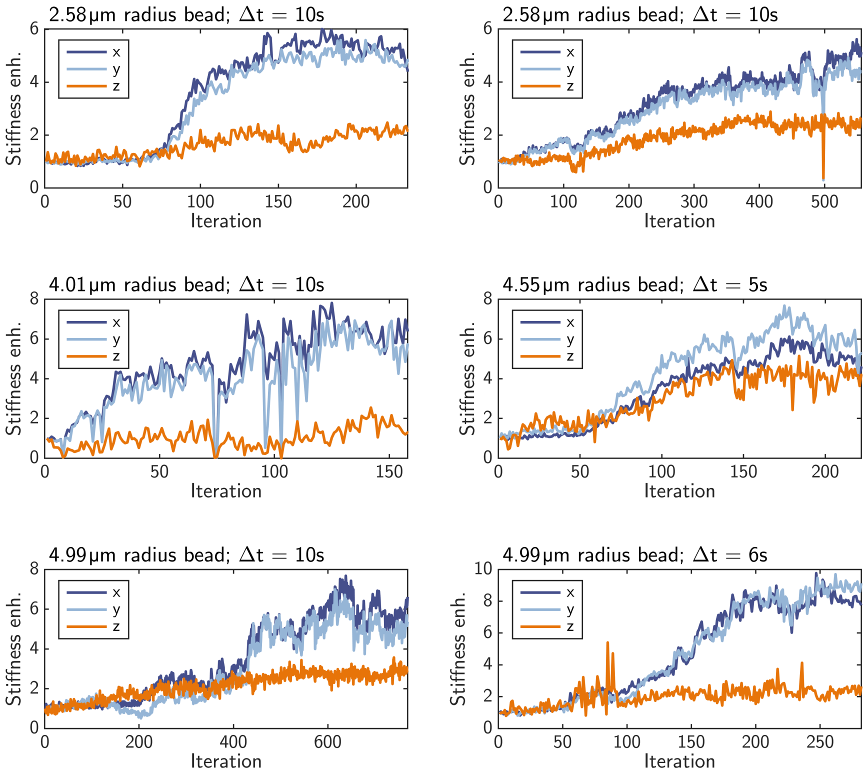

Figure S23: **Examples of the live optimiser convergence behaviour.** Δt is the integration time for estimating the stiffness (see Methods in the main text).

S24. NOISE IN THE LIVE OPTIMISER

To accurately determine the stiffness during live-optimisation one needs to accumulate enough data on particle's motion in order to minimise the effect of noise on optimiser convergence. Here we investigate how the length of the time step Δt during which data is collected affects the measurement noise (see also Methods in the main text).

We first track a trapped particle's centre of mass (CoM) for several minutes. We then break up this data into segments, each of length Δt. We estimate the stiffness for every time segment using the Equipartition theorem (see S25), and then calculate the coefficient of variation (CV) of the stiffness across all segments. We do this analysis for a range of Δt values in order to inform our choice for Δt to be used in a live optimiser.

It can be seen in Fig. S24 that for small time steps ($\Delta t < 5$ s) CV is fairly high, i.e. the measurements of stiffness are very noisy, but the CV stops improving after about 15 s. The exact trend depends on various factors such as particle size, laser power, and temperature. We have found in our experiments that using $\Delta t < 10$ s was a good compromise between levels of noise and optimiser convergence time.

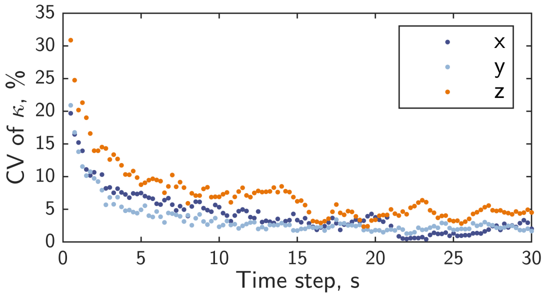

Figure S24: **Coefficient of variation of measured stiffness data**. Data presented here is for a 2.57 μm radius micro-sphere in a conventional optical trap for varying lengths of integration time steps.

When simulating the live optimiser we use the following levels of noise (see also S27):

Bead radius, μm	2.57	4.01	4.55	4.99
$CV_{x,y}$, %	10	10	8	8
CV_z, %	20	20	12	12

S25. SOURCES OF EXPERIMENTAL ERROR

In this section, we detail several sources of experimental errors present in our system.

Random 3D tracking error:
Because our experimental optimisation algorithm relies on accurate 3D tracking of a trapped micro-sphere, the tracking error associated with each measurement of the sphere's displacement needs to be as low as possible. To test the error in our stereoscopic imaging system, we tracked micro-spheres that are fixed to the bottom of our sample. The standard deviation of the tracked displacement of the stationary particle, which we call the error in the tracking of the centre-of-mass (CoM), ϵ_{CoM}, is displayed in the table below for the smallest and largest micro-spheres used. For our experimental setup, we confirm that we are able to track the 3D CoM of stuck micro-spheres with nanometric precision [54].

**Bead radius,** µm	**2.57**	**4.99**
$\epsilon_{\mathrm{CoM}_x}$, nm	0.67	0.51
$\epsilon_{\mathrm{CoM}_y}$, nm	0.73	0.80
$\epsilon_{\mathrm{CoM}_z}$, nm	2.62	2.73

Errors in stiffness enhancement and volume reduction
Here we evaluate errors for the data presented in Fig. 3(d,e) in the main paper. To evaluate stiffness enhancement and volume reduction we track the CoM of the particle in the conventional and optimised traps for several minutes. We then split this data into four segments of equal duration, evaluate the stiffness enhancement in each segment, and take the average. We then evaluate the error in stiffness enhancement as the standard deviation across the four segments – these are shown in Fig. S25. To calculate the stiffness we use the Equipartition theorem, e.g. for the x-dimension we have $\kappa_x = k_{\mathrm{B}}T/\langle x\rangle^2$, where k_{B} is the Boltzmann constant, T is the absolute temperature, and $\langle x\rangle^2$ is the variance of particle's x-coordinate.

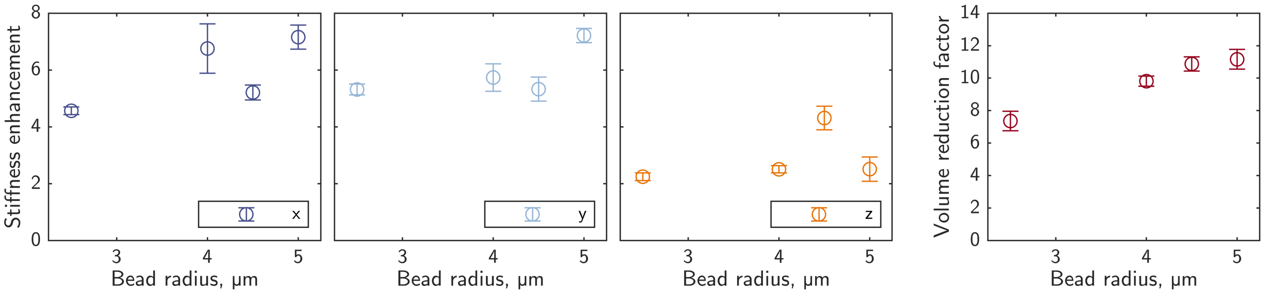

Figure S25: **Error-bars for experimentally achieved stiffness enhancement and confinement volume reduction.** The data presented here is the same as in Fig. 3(d,e) in the main text, but with error-bars included.

We then propagate the stiffness measurement errors through Eqn. 1 in the main text, to evaluate the error on volume reduction. Here we assume that the errors between the three axes are independent and obey a normal distribution. The combined error for the experimentally measured confinement volume V_{c} is then given by:

$$s_{V_{\mathrm{c}}} = \sqrt{\left(\frac{\delta V}{\delta \kappa_x}\right)^2 s^2_{\kappa_x} + \left(\frac{\delta V}{\delta \kappa_y}\right)^2 s^2_{\kappa_y} + \left(\frac{\delta V}{\delta \kappa_z}\right)^2 s^2_{\kappa_z}}. \tag{S34}$$

The partial derivatives are given by $\frac{\delta V}{\delta \kappa_x} = -18\pi\sqrt{\frac{k_{\mathrm{B}}^3 T^3}{\kappa_x^3 \kappa_y \kappa_z}}$, and similarly for κ_y and κ_z. Following from this, the error for the ratio of volumes indicating the volume reduction factor $V_{\mathrm{c}}^{\mathrm{rel}}$ is given by:

$$s_{V_{\mathrm{c}}^{\mathrm{rel}}} = V_{\mathrm{c}}^{\mathrm{rel}} \sqrt{\left(\frac{s_{V_{\mathrm{c}}^{\mathrm{Gauss}}}}{V_{\mathrm{c}}^{\mathrm{Gauss}}}\right)^2 + \left(\frac{s_{V_{\mathrm{c}}^{\mathrm{opt}}}}{V_{\mathrm{c}}^{\mathrm{opt}}}\right)^2}, \tag{S35}$$

where the individual volume errors $s_{V_{\mathrm{c}}^{\mathrm{Gauss}}}$ and $s_{V_{\mathrm{c}}^{\mathrm{opt}}}$ are calculated as above in Eqn. S34.

S26. EFFECT OF TRAP LOCATION ON VOLUME REDUCTION

As we have seen in S20, the performance of pre-designed traps is highly sensitive to particle size and aberrations. We expect that the live-optimised traps will also be susceptible to small perturbations in environment and particle properties. Here we experimentally examine how changing the trap location affects volume reduction.

First, we live-optimise the trapping field, and then translate the trapped particle by ∼18 µm (13 µm in both x and y-directions) from the location at which the trap was optimised. We observe that after translation the confinement volume takes on a slightly different shape – see Fig. S26. As a result the volume reduction has decreased from 10.4 to 6.3. We note that the stiffness of the conventional trap is also reduced by a small amount because of such a translation.

We believe that the confinement volume is influenced by two main factors. Firstly, the trap was translated by adjusting the linear phase ramp displayed on the SLM, which affects the diffraction efficiency and fidelity with which the trapping field is created. A phase ramp with a steeper gradient reduces the SLM diffraction efficiency, hence we observe the conventional trap also suffering reduced stiffness when translated. This reduction in SLM efficiency may not be uniform across the pupil (i.e. being concentrated at phase wrapping lines on the SLM), and so this may alter the shape of the optical field incident on the particle. We expect the 3D optimised trap to be more sensitive to this effect. Secondly, we also expect the physical location within the sample to have an effect on the trap shape, primarily because of subtle variations in thickness of the glass-coverslip and the immersion oil layer changing the aberrations in the beam path to the particle.

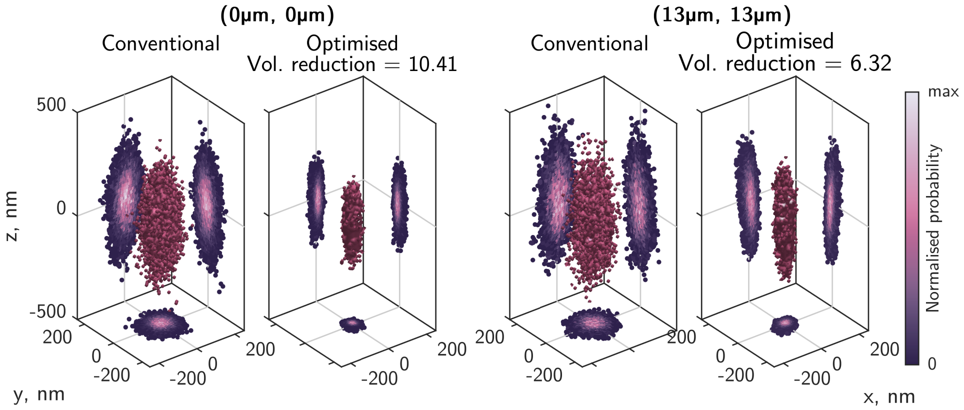

Figure S26: **Effect of trap location on confinement volume reduction.** 3D tracking data for a live-optimised trap at the location of the optimisation (left) and when translated by 13 µm in both x and y (right). The volume reduction quoted here compares the optimised trap to the conventional one at the same location. The z-position in both cases was held constant at 25 µm, and we are trapping a 5 µm radius silica micro-sphere.

S27. EXPERIMENT VS SIMULATIONS

Here we consider in detail how our experimental results compare to simulations. To start with, simulations predict that the highest confinement volume reduction will be achieved with pre-designed traps when full phase, amplitude, and polarisation modulation is available (see Fig. S27). These enhancements are reduced by about 11-21% if polarisation control is removed, and a further 26-40% after removing amplitude control. As discussed in the main manuscript and in S20, the pre-designed traps are not straightforward to implement experimentally, and so we developed a live optimiser, operating in-situ – and basing its decisions on actual *experimental* measurements of stiffness. We also simulated the performance of this live optimiser to better gauge what kind of improvement we can expect to achieve in experiments. In the absence of noise (i.e. if the stiffness could be measured with no error) we expect to see values reaching about 35-75% of the phase-only pre-designed traps, with a further decrease of $\sim$14-37% if realistic levels of noise are taken into consideration (see S24). Finally, our experiments reach $\sim$20-42% of the expected simulated values – a reduction which we believe to originate from residual optical aberrations and SLM diffraction losses which are not present in the simulations.

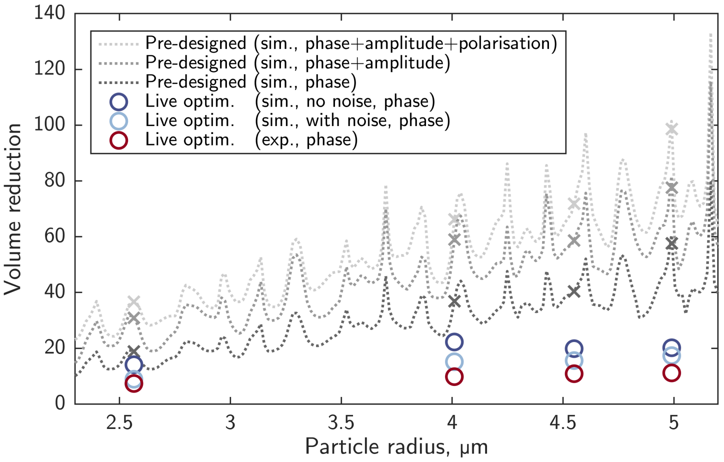

Figure S27: **Comparison of confinement volume reductions achievable in different scenarios**. The crosses overlaid on the grey curves correspond to the experimentally tested bead sizes.

We note that the peaks and valleys observed in the pre-designed volume reduction data are inherited from the Mie-resonances characteristic to conventional optical tweezers (see Fig. S28 pink line), and fluctuations in the optimiser solutions (red line). As seen in Fig. S28, the trend in volume reduction (grey dashed line, which is the ratio of the pink and red lines) is dominated by the Mie-resonances.

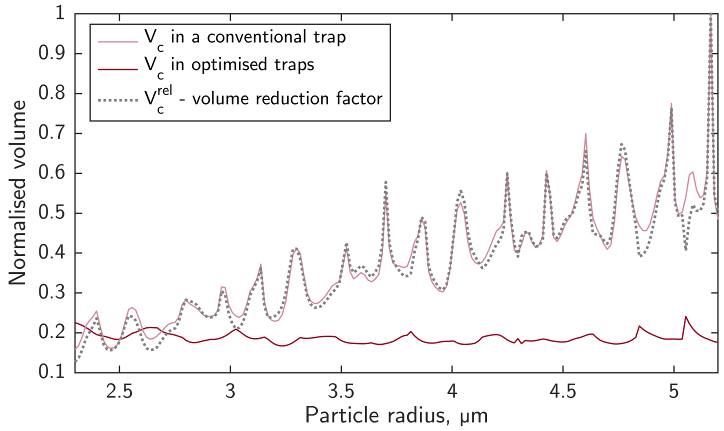

Figure S28: **Resonances and fluctuations in the confinement volume.** Note that the actual values in this figure cannot be compared quantitatively – they have been normalised such that they overlap enough for a qualitative comparison of the trends.

S28. TRAPPING LOW REFRACTIVE INDEX PARTICLES

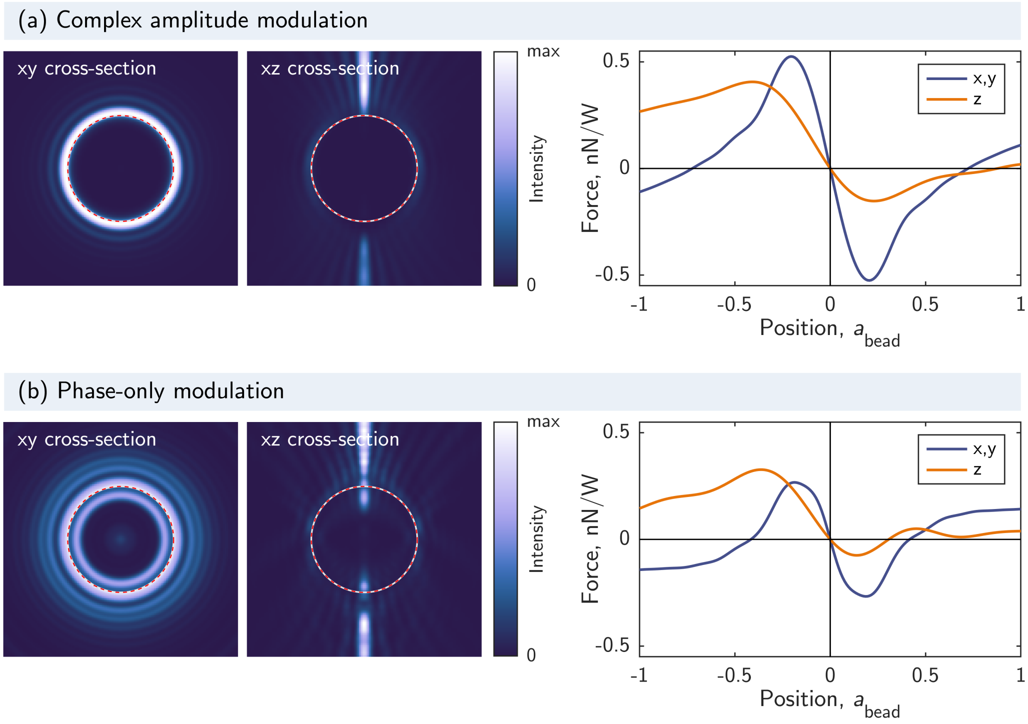

Figure S29: **Low refractive index particle trapping.** Intensity and optical force profiles of traps optimised for a spherical particle with a refractive index lower than the refractive index of the surrounding medium, using (a) full complex amplitude modulation and (b) only phase modulation. The dashed red-white outline indicates the location of the micro-sphere.

Our optimiser can also be used to design beams to trap particles with refractive indices lower than the refractive index of the surrounding medium. In this situation we expect high intensity gradients to reside just outside the particle boundary – as is the case in bottle beams – such that the particle is pushed towards the lowest intensity point. Examples of such beams are presented in Fig. S29, where we model a particle of radius 3 µm and refractive index 1.15, while the surrounding medium has a refractive index of 1.326. The optical force curves clearly indicate a stable trapping point.

S29. TRAPPING RANGE AND MAXIMUM RESTORING FORCE

Optical trapping range and maximum restoring force are important features of optical traps. In the following we define $f_{\max/\min}$ as the maximum/minimum restoring force pulling the trapped particle towards the equilibrium - this is illustrated in Fig. S30. We note that some optimised traps have multiple local force maxima and minima, as seen for example in Fig. 1D - here we always choose the one closest to the equilibrium. Then we define the distance between the equilibrium and the maximum/minimum restoring force as the trap range $R_{\text{left/right}}$. For both conventional and 3D-optimised traps in the transverse directions x and y we have: $f_{\max} = f_{\min}$ and $R_{\text{left}} = R_{\text{right}}$, but the symmetry is broken in the axial direction. Figures S31 and S32 show the trap range and restoring forces for the conventional and our optimised traps, as well as the ratio of these quantities.

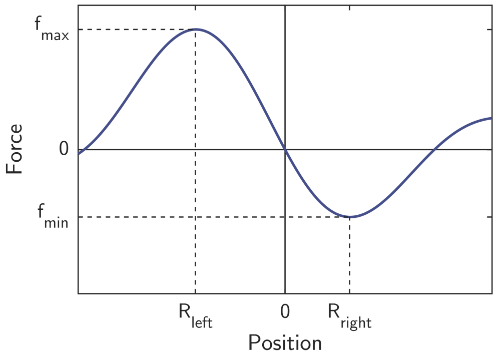

Figure S30: **Schematic of a force profile indicating the trapping range R and maximum restoring force f.**

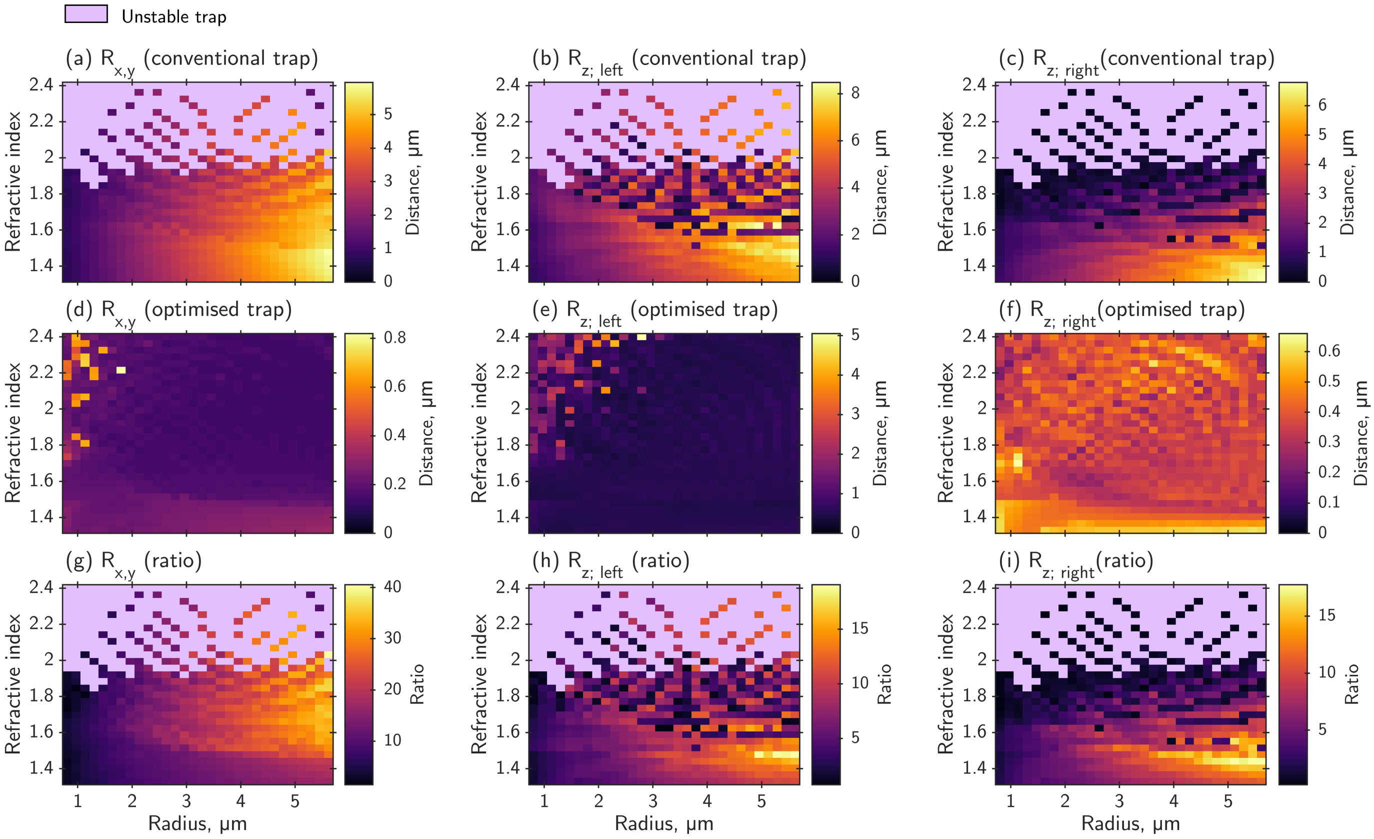

Figure S31: **Comparison of trap range in conventional and 3D-optimised traps.** The first row shows the following for a conventional optical trap: (a) the trap range along the x and y directions, (b) the left trap range along the z direction, and (c) the right trap range along the z direction. The second row shows the same but for the 3D-optimised traps, and the last row shows the ratio between the two (conventional over optimised).

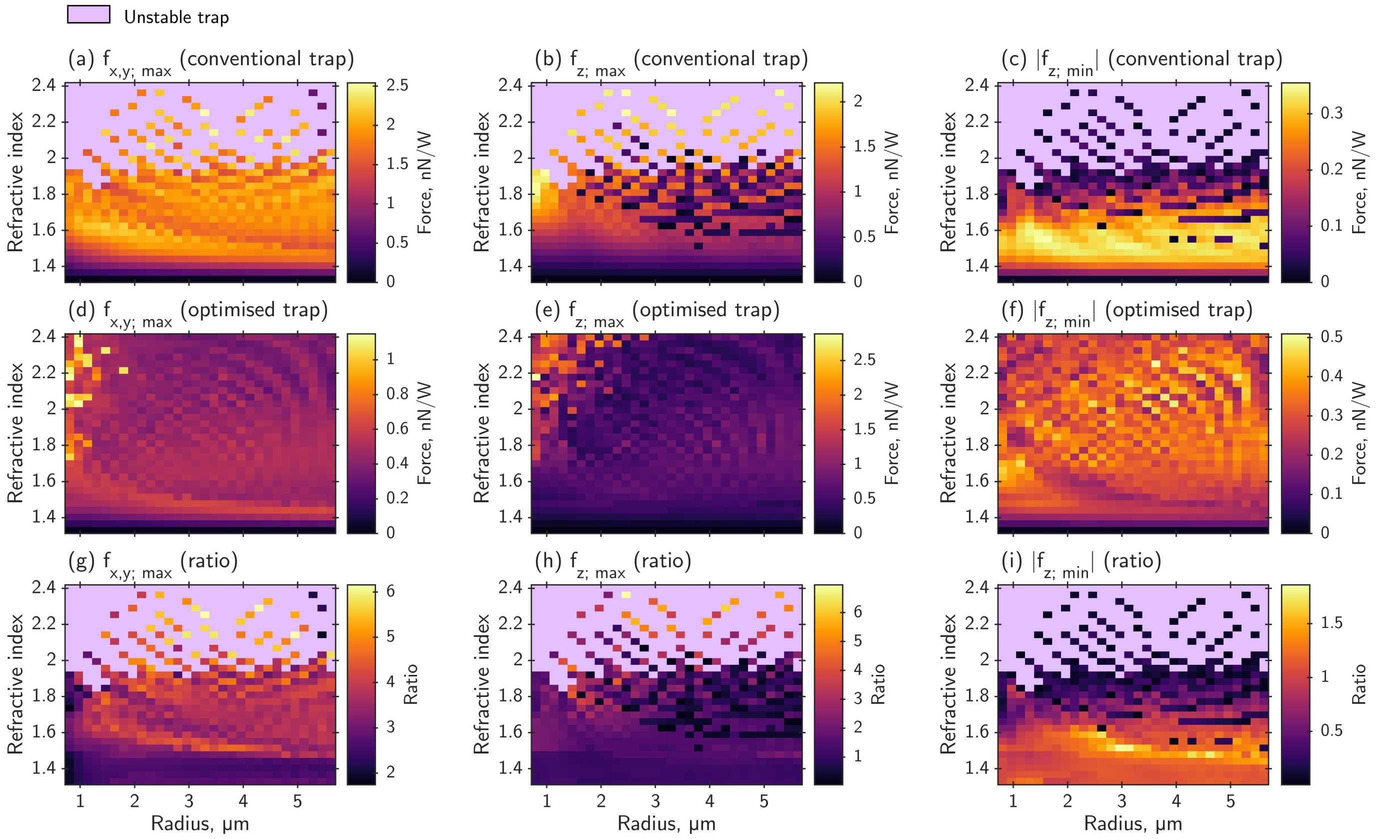

Figure S32: **Comparison of maximum restoring forces in conventional and 3D-optimised traps.** The first row shows the following for a conventional optical trap: (a) the maximum restoring force along the x and y directions, (b) the maximum positive restoring force along the z direction, and (c) the absolute value of the most negative restoring force along the z direction. The second row shows the same but for the 3D-optimised traps, and the last row shows the ratio between the two (conventional over optimised).

S30. STABILITY OF OPTIMISED TRAPS AGAINST THERMAL PERTURBATIONS

The very high stiffness of our optimised traps comes at the expense of reduced peak restoring forces and effective trap range. It is therefore pertinent to consider the minimum optical power required to ensure optical forces are high enough to prevent thermal perturbations from ‘kicking’ the particle out of the trap.

We do this by numerically solving the Langevin equation in the non-inertial regime to simulate the motion of the particle [2], and observing if it leaves the ‘stable trapping region’. We define the bounds of this stable trapping region as the locations of the optical force minimum/maximum nearest to the trap equilibrium (for several different directions in order to obtain a 3D region). If there is no nearby minimum/maximum, we take the particle radius as the bound. We repeatedly solve the Langevin equation for different beam powers until we find the power at which the particle never crosses the defined trap boundary over a 5 min period. Once the minimum power is identified, we simulate 12 additional 5 min trajectories to confirm that the particle indeed does not escape the trap.

Based on these simulations we estimate that for the vast majority of our traps several mW of power are enough to ensure thermal stability, which is typical for standard trapping experiments. More power (in the region of low-tens of mW) might be needed for traps of small particles with very high refractive indices, since the restoring z-force for some of these traps can be quite small.

S31. USING OAM BEAMS IN THE LIVE OPTIMISER

Here we explore if the live optimiser might benefit from using light carrying OAM. We introduce OAM by allowing the phase of every ring to wrap from 0 to 2π along the perimeter of the ring a set number of times. Figure S33 showcases results for two particles of different sizes. For the small particle with 2 µm radius introduction of OAM immediately worsens the results. This is because the light emanating from centre-most rings in the far field misses the particle, thus reducing the power available for creating the trap. For the larger 4 µm radius particle, we do not see any improvement for OAM values up to 13; anything larger than that caused trap instabilities.

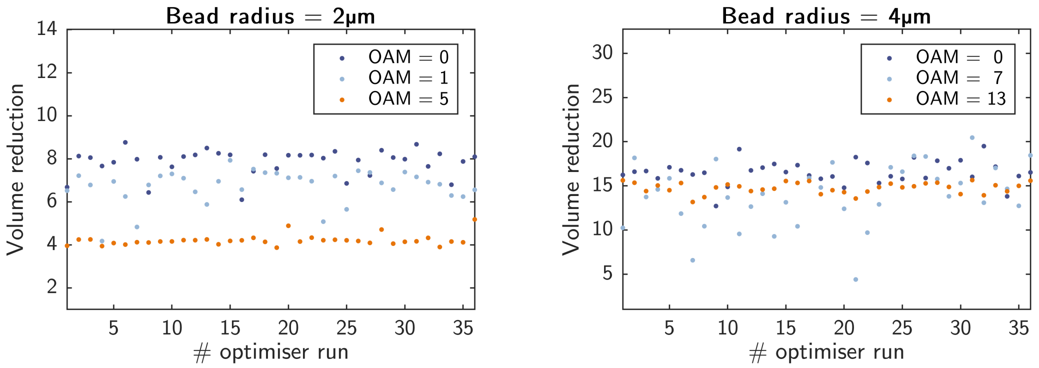

Figure S33: **Volume reduction achieved with the live optimiser (in a noiseless simulation) when different values of OAM are used.** Here we simulated silica particles ($n = 1.45$), immersed in water ($n = 1.326$), and illuminated with a circularly polarised 1064 nm beam, through NA of 1.13.

It is worth noting that in these simulations every ring was assigned the same OAM value. It might instead be beneficial to set different values to different rings in an effort to favourably structure the light surrounding the particle. It is not, however, immediately apparent what the best distribution of OAM values would be. The maximum reasonable OAM value to be used for each far-field ring could be deduced by considering whether the intensity ring formed in the object plane overlaps with the particle. One might then envision adapting the optimisation routine to include OAM values as optimisation variables alongside the global phases of the rings. Such modification would undoubtedly increase the complexity of the optimisation, and may be something to be explored in the future.

S32. SUPPLEMENTARY MOVIE

Movie S1. Examples of pre-designed optical traps. These traps were optimised for the following particles (from left to right): 1) radius: 4.01 µm, refractive index: 1.45, circular polarisation, phase+amplitude modulation, confinement volume reduction: 98.75; 2) radius: 2.96 µm, refractive index: 1.33, linear polarisation, phase+amplitude modulation, confinement volume reduction: 45.84; 3) radius: 5.28 µm, refractive index: 1.66, linear polarisation, phase+amplitude modulation, confinement volume reduction: 56.97; 4) radius: 2.79 µm, refractive index: 1.37, circular polarisation, phase modulation, confinement volume reduction: 34.10; 5) radius: 2.13 µm, refractive index: 2.18, phase+amplitude+polarisation modulation, corresponding conventional trap is unstable. Other parameters are the same as listed in Fig. 1 in the main text.
